# Structural insights into CodY activation and DNA recognition

**DOI:** 10.1093/nar/gkad512

**Published:** 2023-06-16

**Authors:** Tobias Hainzl, Mari Bonde, Fredrik Almqvist, Jörgen Johansson, A Elisabeth Sauer-Eriksson

**Affiliations:** Department of Chemistry, Umeå University, 901 87 Umeå, Sweden; Umeå Centre of Microbial Research (UCMR), Umeå University, Umeå, Sweden; Department of Chemistry, Umeå University, 901 87 Umeå, Sweden; QureTech Bio, Umeå, Sweden; Department of Chemistry, Umeå University, 901 87 Umeå, Sweden; Umeå Centre of Microbial Research (UCMR), Umeå University, Umeå, Sweden; Umeå Centre of Microbial Research (UCMR), Umeå University, Umeå, Sweden; Department of Molecular Biology, Umeå University, 901 87 Umeå, Sweden; Molecular Infection Medicine, Sweden (MIMS), Umeå University, 901 87 Umeå, Sweden; Department of Chemistry, Umeå University, 901 87 Umeå, Sweden; Umeå Centre of Microbial Research (UCMR), Umeå University, Umeå, Sweden

## Abstract

Virulence factors enable pathogenic bacteria to infect host cells, establish infection, and contribute to disease progressions. In Gram-positive pathogens such as *Staphylococcus aureus* (*Sa*) and *Enterococcus faecalis* (*Ef*), the pleiotropic transcription factor CodY plays a key role in integrating metabolism and virulence factor expression. However, to date, the structural mechanisms of CodY activation and DNA recognition are not understood. Here, we report the crystal structures of CodY from *Sa* and *Ef* in their ligand-free form and their ligand-bound form complexed with DNA. Binding of the ligands—branched chain amino acids and GTP—induces conformational changes in the form of helical shifts that propagate to the homodimer interface and reorient the linker helices and DNA binding domains. DNA binding is mediated by a non-canonical recognition mechanism dictated by DNA shape readout. Furthermore, two CodY dimers bind to two overlapping binding sites in a highly cooperative manner facilitated by cross-dimer interactions and minor groove deformation. Our structural and biochemical data explain how CodY can bind a wide range of substrates, a hallmark of many pleiotropic transcription factors. These data contribute to a better understanding of the mechanisms underlying virulence activation in important human pathogens.

## Graphical Abstract

**Graphical Abstract:**
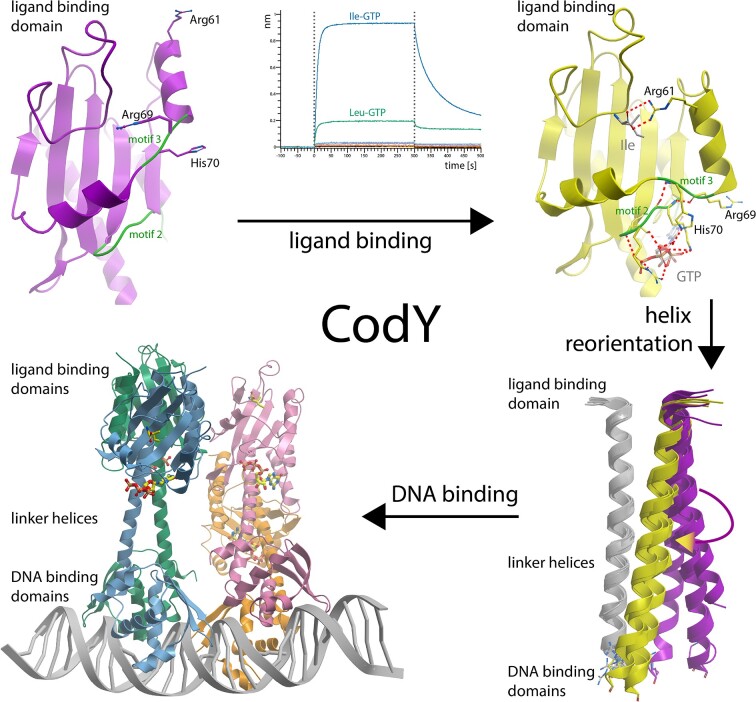

## INTRODUCTION

*Staphylococcus aureus* (*Sa*) and *Enterococcus faecalis* (*Ef*) are two Gram-positive bacteria that can reside in humans commensally or as pathogens able to cause life-threatening infections. The transition between their commensal and pathogenic states is controlled by a complex network of transcription factors which adjust gene expression and ensure adaptation to diverse host environments. The pleiotropic transcription factor protein CodY is a global transcription factor, found in almost all low G + C Gram-positive bacteria. In these bacteria, CodY functions as a regulator of transcription for several hundred genes. The majority of CodY target genes encode for proteins involved in metabolic pathways; however, CodY also directly or indirectly controls the expression of essential virulence factors (1–10). CodY is therefore thought to act as a regulatory link between metabolism and virulence, meaning it plays a key role in the transition between the commensal and pathogenic states (5,7,10). To date, the molecular mechanisms underlying CodY activation and target gene recognition remain poorly understood.

CodY DNA-binding is activated by two types of ligands: branched chain amino acids (BCAAs, i.e. isoleucine, leucine, and valine) and, in most cases, guanosine triphosphate, GTP (11–14). CodY from Bacilli, Clostridia, Listeria and Staphylococci responds to both BCAAs and GTP, whereas CodY from Enterococci, Streptococci and Lactococci respond to BCAAs but not to GTP (15,16). In essence, CodY monitors the metabolic and energetic status of the cell by sensing the levels of BCAAs and GTP. In most cases, CodY acts as a repressor by binding to DNA and blocking binding of RNA polymerase and transcriptional activators, or by prematurely terminating transcription via a roadblock mechanism. Therefore, genes under CodY-control are mainly repressed in nutrient-rich environments when intracellular levels of BCAAs and GTP are high (1,3,17).

The structure of the CodY homodimer is known (15,18). Each 30 kDa protomer comprises the N-terminal ligand-binding domain called GAF (c**G**MP-specific phosphodiesterases, **a**denylyl cyclases and **F**hlA). The GAF domain is connected by an extended linker helix to the C-terminal DNA-binding (DBD) domain that contains a winged helix-turn-helix (wHTH) motif. Ligand-binding in the GAF domains is proposed to re-orient the linker helices that position the DBD domains for DNA-interaction (15,18). The DNA-binding consensus sequence for CodY has been identified as the 15-nucleotide (nt) pseudo-palindromic sequence AATTTTCWGAAAATT (W is A or T) (19). Genome-wide analysis of *Sa*, *Bacillus subtilis* (*Bs*), *Clostridium difficile* and *Listeria monocytogenes* reveals that more than one hundred direct CodY-binding sites exist (20–23). These are located in regulatory noncoding regions but, to a large degree, also within coding regions. Notably, CodY-binding sites show a high degree of sequence variability, giving rise to a wide range of CodY affinities. Sites with even five mismatches to the consensus sequence play important roles in CodY-mediated regulation (24). Owing to the high sequence variation of CodY-binding sites, threshold concentrations of active CodY required to trigger a transcriptional response vary among CodY target genes and allow for a finely tuned transcriptional response to diverse BCAA- and GTP-levels (25).

Interestingly, as first reported for *Bs*, CodY-regulatory regions typically contain two copies of the 15-nt binding sequence that overlap with 6-nt to form a 24-nt one (24). Genome-wide analysis has revealed that such overlapping binding sites are conserved in CodY-regulatory regions of other Gram-positive bacteria (20–23). This raises the possibility that CodY-dependent genes largely rely on overlapping binding sites for regulation. Moreover, binding of CodY to overlapping binding sites is highly cooperative (24). Cooperative binding of CodY to DNA thus adds an additional layer of transcriptional complexity that appears to play an important role in CodY function.

The key role of CodY in the transition between commensal and pathogenic states warrants a detailed understanding of the molecular mechanisms underlying CodY-dependent gene regulation. Structural analysis of free- and ligand-bound CodY from *Bs, B. cereus* (*Bc*) and *Sa* has provided important insights into the mechanism of BCAA and GTP binding and ligand-induced domain movements (15,18). However, the link between ligand binding and DNA-binding remains poorly understood, and above all, the basis of CodY target-site recognition and cooperativity is not known. Here, we report biochemical data and X-ray structures of CodY from *Sa* and *Ef* (referred to as SaCodY and EfCodY) in ligand-free form and in complex with a 30-nt DNA consensus sequence comprising two 15-nt binding sequences with a 6-nt overlap. Our data illuminate the ligand-induced structural changes that govern CodY activity and explain how cooperative DNA binding enables two CodY dimers to recognize overlapping binding sites of highly variable sequence. Our analysis highlights mechanistic similarities and differences between CodY proteins and provides a stepping-stone for therapeutic targeting of the virulence regulator CodY.

## MATERIALS AND METHODS

### Cloning, expression and purification

The full-length SaCodY and EfCodY coding sequences (UniProt Q2FHI3 and Q834K5) were PCR-amplified from genomic DNA and ligated into a pETHis_1a vector (26) using the Nco1 and Acc651 restriction sites. Mutations in the coding sequences were generated using overlap extension PCR. All constructs were verified by DNA sequencing. For CodY overexpression, the pETHis_1a-CodY vectors were transformed into *Escherichia coli* strain BL21(DE3)pLysS. An overnight culture of the transformed strain in LB medium was diluted 1/100 and cultured at 37°C until a cell density of 0.4–0.6 at OD_600_. At this density, the temperature was reduced to 18°C and CodY expression was induced with 0.5 mM isopropyl β-d-1-thiogalactopyranoside (IPTG). After IPTG addition, the cells were further cultured for ∼16 h at 18°C before harvest. After cell-lysis by sonication, CodY was captured from the supernatant on Ni-NTA (Qiagen). After elution with imidazole, CodY was incubated for ∼12 h at 4°C with Tobacco Etch Virus (TEV) protease (100:1 molar ratio) to cleave the N-terminal His_6_-tag. The cleavage reaction—dialyzed with a 15-kDa cutoff membrane against imidazole-free buffer—was reloaded onto Ni-NTA to capture uncleaved CodY and the His_6_-tagged TEV protease. The flow-through contained cleaved, full-length CodY with a glycine and alanine residue—remnants of the TEV cleavage site preceding the start methionine residue. CodY was further purified by size exclusion chromatography on a Superdex S200 column (Cytiva). Note that the NaCl salt concentration during the entire CodY purification procedure was kept at a minimum of 200 mM to prevent CodY aggregation. SaCodY was concentrated to 360 μM (the given molarities refer to the CodY protomer concentrations) in the Superdex S200 buffer containing 20 mM Tris–HCl pH 8, 200 mM NaCl. EfCodY was concentrated to 690 μM in the Superdex S200 buffer containing 20 mM Tris–HCl pH 8, 500 mM NaCl.

### Bio-layer interferometry

5′-Biotinylated DNA-oligos (Eurofins) were annealed with non-biotinylated complementary DNA-oligos by mixing them 1:1.1, heating the mixture for 5 min in boiling water, and slow cooling. 100 nM dsDNA—containing a 3-nt single-stranded overhang at the biotinylated 5′-end—was captured on Streptavidin (SA) biosensors of the Octet system (Sartorius). The loaded biosensors were subsequently incubated at 25°C and a shaking speed of 1000 rpm with different concentrations of CodY in a buffer containing 20 mM Tris–HCl pH 8, 150 mM NaCl, 2 mM MgCl_2_ and 0.1% NP-40. 2 mM GTP and 10 mM of either Ile, Leu or Val was added singly or in combination to the buffer in separate experiments performed in independent triplicates. The times for the base line, association, and dissociation steps were 300, 600 and 600 s, respectively. CodY-DNA binding and dissociation was measured in real-time. After control and reference subtraction, as well as base line alignment, kinetic parameters were analysed using the Octet Analysis Studio Software. The binding curve data of twofold CodY-dilution series and a 2:1 interaction model for global curve fitting were used to determine the dissociation constant (KD). The KD at equilibrium was calculated with the steady state equation:


$$\begin{equation*}{\rm{Response}} = {{\rm{R}}_{{\rm{max}}}}{\rm{x Conc}}/\left( {{\rm{KD}} + {\rm{Conc}}} \right){\rm{.}}\end{equation*}$$

### Mass photometry

Mass photometry analysis was performed using a Refeyn OneMP mass photometer (Refeyn Ltd). Movies of 6000 frames (60 s) were acquired using AcquireMP software with default settings. Briefly, contrast-to-mass calibration was performed using a native-marker protein standard mixture composed of eight proteins from 20 to 1200 kDa (NativeMark Unstained Protein Standard, Thermo Fisher) in phosphate-buffered saline (PBS). Prior to the measurements, the objective was focused on the surface of the glass–buffer interface with 8 μl of 20 mM Tris–HCl pH 8, 200 mM NaCl. Movies were acquired after addition of 8 μl of 100 nM CodY-proteins in the same buffers. The recorded data were processed using DiscoverMP software (Refeyn Ltd). The data were plotted as mass distribution histograms, and the distribution peaks were fitted with Gaussian functions to obtain the average molecular mass.

### Size-exclusion chromatography coupled to multi-angle light scattering (SEC-MALS)

SEC-MALS was performed using an ÄKTApure system (GE Healthcare) coupled to a miniDAWN TREOS II detector and an OptiLab T-rEX online refractive index detector (Wyatt Technology). The absolute molar mass was calculated by analysing the scattering data with the ASTRA analysis software package, version 7.2.2.10 (Wyatt Technology). Bovine serum albumin (BSA) was used for calibration, and proteins were separated on a Superdex 75 10/300 analytical column (GE Healthcare) at a flow rate of 0.4 ml/min. CodY (200 μl of 35 μM) was injected and eluted in 20 mM Tris–HCl pH 8, 200 mM NaCl. The refractive index increment was set at 17.66 μM for EfCodY and 5.37 μM for SaCodY, and the extinction coefficient for ultraviolet detection at 280 nm was calculated from the primary sequences.

### Crystallization

Crystals were grown by sitting-drop vapour diffusion at 18°C and appeared within 2–10 days. For crystallization of ligand-free CodY, SaCodY (270 μM) in 150 mM NaCl was mixed 1:1 with 0.2 M ammonium sulphate, 0.1 M sodium acetate pH 4.6, and 25% (w/v) polyethylene glycol (PEG) 4000. Crystals were cryo-protected by a brief soak in mother liquor supplemented with 35% (w/v) PEG 4000. EfCodY (600 μM) in 450 mM sodium chloride was mixed 1.3:1 with 0.2 M ammonium phosphate, 2.5% ethanol and 23% (w/v) PEG 3350. Crystals were cryo-protected by a brief soak in mother liquor supplemented with 35% (w/v) PEG 3350. For crystallization of the CodY–DNA complexes, the DNAs were prepared as follows: DNA-oligos (Eurofins) were dissolved in 10 mM Tris–HCl pH 8, 50 mM NaCl, and 1 mM ethylenediaminetetraacetic acid (EDTA) to a final concentration of 0.4 mM. Complementary DNA-oligos were mixed 1:1 and annealed by placing the mixture for 5 min in boiling water, followed by slow cooling. The CodY–DNA complexes were prepared by mixing CodY with DNA at a molar ratio of 4:1 to final concentrations of 140 μM and 35 μM (2 mM GTP, 20 mM Ile) for the *Sa*-complex, and 200 μM and 60 μM (4 mM Leu) for the *Ef*-complex. The mixtures—with final NaCl concentrations of 150 mM—were incubated for at least for 4 h at room temperature (RT). Several DNAs from 28 to 32 base pairs (bp) in length with different 5′- and 3′-termini (blunt-ended or sticky-ended) containing sequence-optimized overlapping binding sites (24) were used for crystallization trials. Diffracting crystals for both SaCodY and EfCodY were obtained with a blunt-ended 30-bp DNA (5′-GAT*AATTTTCAGAATTTTCAGAAAATT*TAG-3′; CodY consensus sequence is highlighted in italics). For this, the SaCodY-DNA complex was mixed 1.5:1 with 0.15 M ammonium sulphate, 0.1 M MES pH 5.4, and 25.5% (w/v) PEG 4000. Crystals were cryo-protected by a brief soak in mother liquor supplemented with 35% (w/v) PEG 4000. The EfCodY–DNA complex was mixed 2:1 with 0.01 M cobalt chloride, 0.01 M manganese chloride, 0.1 M sodium acetate pH 4.6, and 1 M 1,6-hexanediol. Crystals were cryo-protected by a brief soak in mother liquor supplemented with 20% glycerol.

### Structure determination, model building and refinement

Diffraction data were collected at 100 K at the MAX IV synchrotron in Lund (SaCodY, beamline Biomax, λ = 0.9762 Å) and the European Synchrotron Radiation Facility in Grenoble (SaCodY-Ile-GTP-DNA, beamlines ID23-2, λ = 0.8731 Å; EfCodY, ID30B, λ = 0.9763 Å; and EfCodY-Leu-DNA, ID30B, λ = 0.9116 Å). Diffraction images were processed with XDS (27) and scaled and merged using AIMLESS from the CCP4 software suite (28). All structures were determined by molecular replacement with the program PHASER from the PHENIX program suite (29) using the ligand-bound SaCodY structure, PDB code 5ey1 (15) as the initial search model. The atomic models were manually built using the program COOT (30) and refined with PHENIX Refine (29) using non-crystallographic symmetry (NCS) restraints (31). Each chain of the pseudo-palindromic DNA is numbered from –2 to + 27. Note that crystal packing interactions in the SaCodY- and EfCodY-DNA complexes did not involve linker helices and DBD domains. Interface residues and nucleotides were well defined in the electron density, but at the present resolution, we were unable to confidently resolve interfacial water molecules. Data collection and refinement statistics are shown in Supplementary Table S1. The diffraction data of the SaCodY-Ile-GTP complex with DNA were processed and refined in space group *P*6_1_22. The asymmetric unit consisted of one SaCodY dimer and one ssDNA comprising both DNA strands, refined with half occupancy. The asymmetric unit of the crystal and the biological assembly of the SaCodY-Ile-GTP complex with DNA are shown in Supplementary Figure S1. POLDER maps (32) were used to verify Ile and GTP binding to protomers A and B in the SaCodY-DNA complex structure, and Leu-binding to protomers A and B in the EfCodY-DNA complex structure. For all ligands, the polder map was likely to show the omitted atoms (CC(1,3) values >0.75, Supplementary Figure S2. GTP binding was better defined in the electron density of protomers A and C than in protomers B and D. The following residues could not be modelled due to lack of density, suggesting that these residues are flexible: ligand-free EfCodY, Lys260 in chain A, Val23-Glu26 in chain C, and Asn182-Lys260 in chain C. Furthermore, almost all side chains within the DBD domain of protomer B were poorly defined resulting in a high number of RSRZ outliers: EfCodY-Leu-DNA, Asn18-Val23 in chain C. The DNA inter-strand phosphate–phosphate distances were calculated using the 3DNA program (33). Figures were prepared with ICM browser (https://www.molsoft.com) and CCP4mg (34).

## RESULTS

### Leucine promotes EfCodY DNA binding

**Figure 1.**
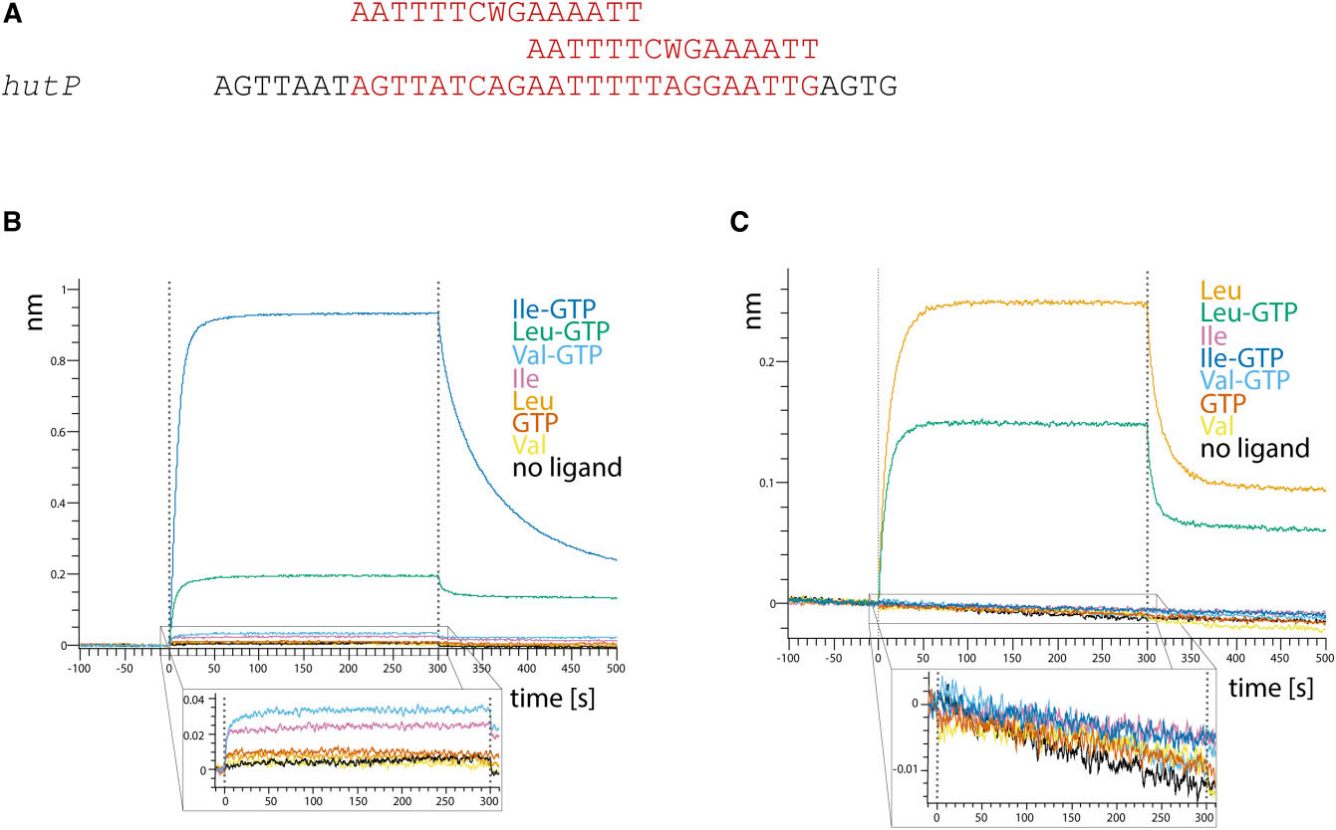
SaCodY and EfCodY respond differently to BCAAs and GTP. (**A**) Sequence of the overlapping binding sites in the *hutP* operator of *Bs* used in the bio-layer interferometry assay. Binding sites are highlighted in red; the consensus sequence for the two single sites is shown above the *hutP* operator sequence. (**B**) GTP and Ile activate SaCodY synergistically. Representative sensorgrams of the interaction of 1 μM SaCodY with the *hutP* operator sequence in absence or presence of 2 mM GTP and 10 mM of either Ile, Leu or Val added singly or in combination. Strong association signals—indicating increased DNA affinity of SaCodY—are observed only in presence of both GTP and Ile (Leu). (**C**) Leu but not Ile activates EfCodY. Representative sensorgrams of the interaction of 1 μM EfCodY with the *hutP* operator sequence. Strong signals are only observed in presence of Leu. Note that GTP (but not ATP, CTP, TTP and GDP, data not shown) exerts a negative effect on Leu-activation of EfCodY. The insets in (B) and (C) show an enlarged view of the association step for the low signal sensorgrams.

To gain a better understanding of how ligand binding promotes DNA binding, we used bio-layer interferometry. For this, biotinylated DNA substrates were immobilised on streptavidin biosensors and incubated with CodY in solution to analyse the affinity of the interaction. As a DNA substrate we used the DNA sequence of the well-characterized overlapping CodY-binding sites in the *hutP* operator of *Bs* (24). For comparison, we analysed the affinity of both SaCodY and EfCodY in the absence and presence of the ligands BCAAs and GTP (Figure 1). The analysis showed that SaCodY was activated by Ile (and to a lesser extent by Leu) and GTP as expected, but only if both were present simultaneously. Furthermore, we uncovered that—in contrast to SaCodY—only Leu activated DNA binding of EfCodY. Consistent with prior predictions based on sequence homology (15,16), GTP did not activate EfCodY. Together with Leu, GTP reduced activation, presumably by interacting with the protein and preventing Leu-induced conformational changes.

### Ligand-free SaCodY is monomeric and ligand-free EfCodY is dimeric

**Figure 2.**
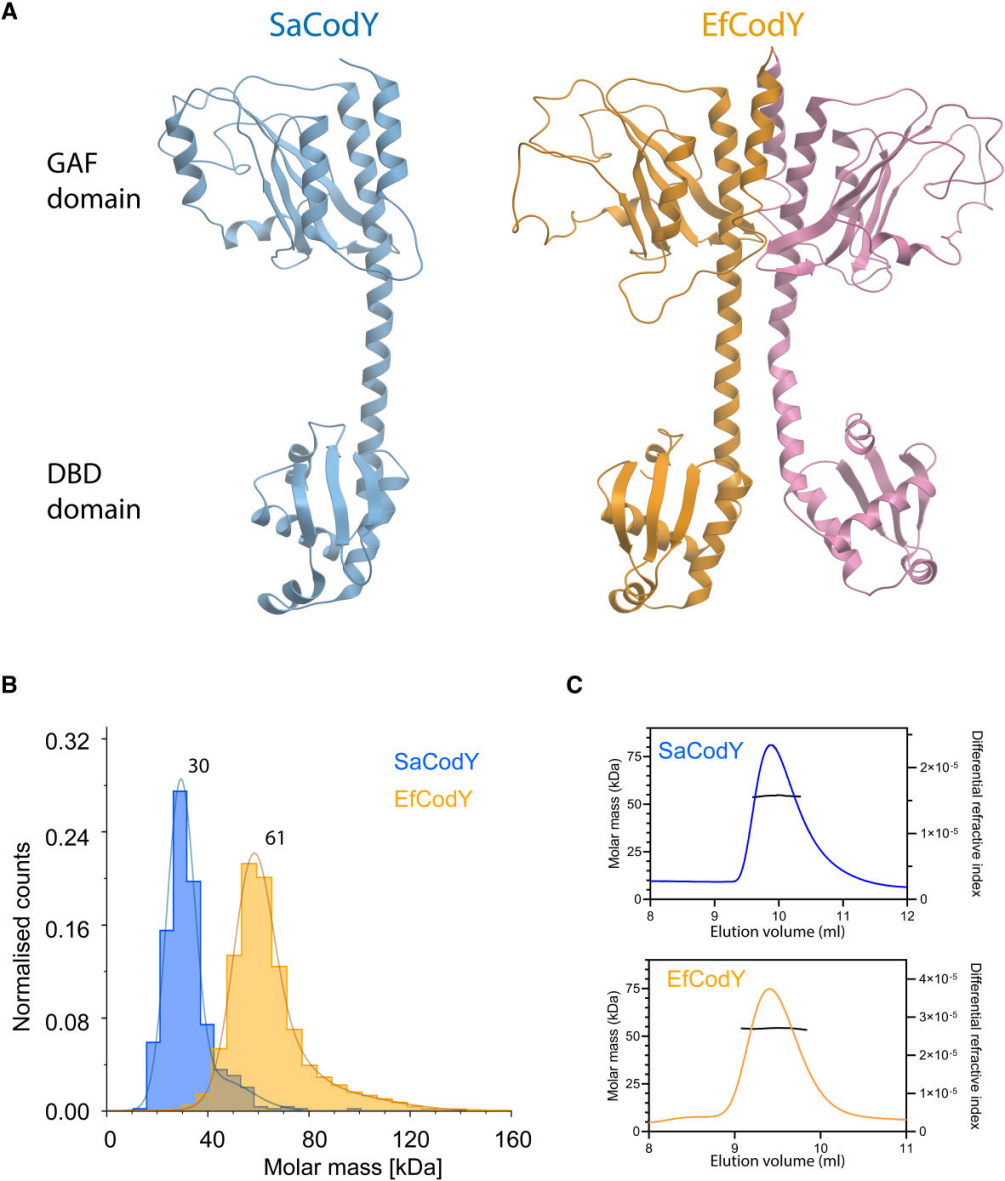
Overall structure of ligand-free SaCodY and EfCodY. (**A**) Ribbon representation of the ligand-free monomeric SaCodY (left, monomer A) and dimeric EfCodY (right, protomers A and B) crystal structures. (**B**) Ligand-free SaCodY at 100 nM concentration is monomeric in solution as confirmed by mass photometry. Molecular mass distribution histograms of ligand-free SaCodY (blue) and EfCodY (orange). The average molecular masses agree well with the theoretical masses of 28.9 kDa for monomeric SaCodY and 58.1 kDa for dimeric EfCodY. (**C**) At high concentration SaCodY forms dimers in solution as confirmed by SEC-MALS. SEC-MALS profiles of SaCodY and EfCodY at 35 μM concentration. The measured masses of 54.3 kDa for SaCodY and 54.1 kDa for EfCodY agree with theoretical masses of 57.8 kDa for dimeric SaCodY and 58.1 kDa for dimeric EfCodY.

Next, we solved the crystal structures of ligand-free SaCodY and EfCodY. Both structures are similar to previously reported CodY structures in that they have physically separated GAF and DBD domains connected by the extended linker helix (15,18). Unexpectedly however, the structures revealed a previously undescribed monomeric form of SaCodY and a dimeric EfCodY (Figure 2A). Mass photometry analysis of ligand-free SaCodY and EfCodY in solution supported the oligomerization states observed in our crystal structures. Nevertheless, ligand-free SaCodY can also form dimers, as confirmed with SEC-MALS (Figure 2B, C). This suggests that a monomer–dimer equilibrium may be a factor in regulation of SaCodY activity. The previously reported crystal structures of ligand-free BsCodY (18) and BcCodY (15) are tetrameric CodY, but we found no evidence for tetrameric forms of SaCodY and EfCodY in our mass photometry or SEC-MALS experiments. This is in line with prior suggestions that the tetrameric form in the crystal lattice of ligand-free BsCodY is caused by the high CodY concentrations used for crystallization. In other words, the tetramer-interface is probably an artefact of the crystallization (18).

The GAF domains of the ligand-free EfCodY protomers pack with close to perfect 2-fold symmetry. However, this symmetry breaks down in the C-terminal part of the linker helices as well as the DBD domains, which all assume different orientations in the four protomers in the asymmetric unit. Furthermore, for protomer chain C, parts of the linker helix and the DBD domain could not be modelled (residues 181–260). This evidenced the flexibility of them in the ligand-free EfCodY, in consistency with previous structures of CodY (15). The asymmetric unit of the ligand-free SaCodY crystals contains two well-structured monomers with their GAF and DBD domains tightly packed head-to-tail (Supplementary Figure S3AB). Interestingly, the buried surface area in the head-to-tail packing of two ligand-free SaCodY monomers in the crystal is similar in size to the buried surface area of two protomers in the Ile/GTP-bound dimer (∼1600 Å^2^, PDB code 5ey0 (15)). Therefore, it is possible that the dimer observed in the SEC-MALS experiments of ligand-free SaCodY (Figure 2C) is the non-biological head-to-tail dimer observed in the crystals. To determine if the DBD domain of ligand-free SaCodY is flexible in solution, we used hydrogen deuterium exchange mass spectrometry, which did show the DBD domain to be flexible (Supplementary Table S2 and Supplementary Figure S3CD).

### Ligand binding induces a conformational change that is propagated to the linker helix

The GAF domain consists of a five-stranded ß-sheet (S1–S5), packed on the inner face against a 3-helix bundle (H1, H2, and the N-terminal part of the linker helix). The outer face of the ß-sheet is packed against a more irregular structure of two long loops comprising α-helices H3 and H4. In the structure of the Ile/GTP-bound SaCodY, the GTP is located close to the dimer interface. Two residues from the linker helix, and six residues from three regions: the two loops connecting H1-H2 (motif 1) and S1–S2 (motif 2), as well as the H3–H4 linker (motif 3) tightly interact with the GTP molecule (15). In contrast, the Ile binding site is distant from the dimer interface; it is located on the outer face of the ß-sheet, where two long loops including α-helices H3 and H4 wrap around the Ile molecule (15,18,35).

**Figure 3.**
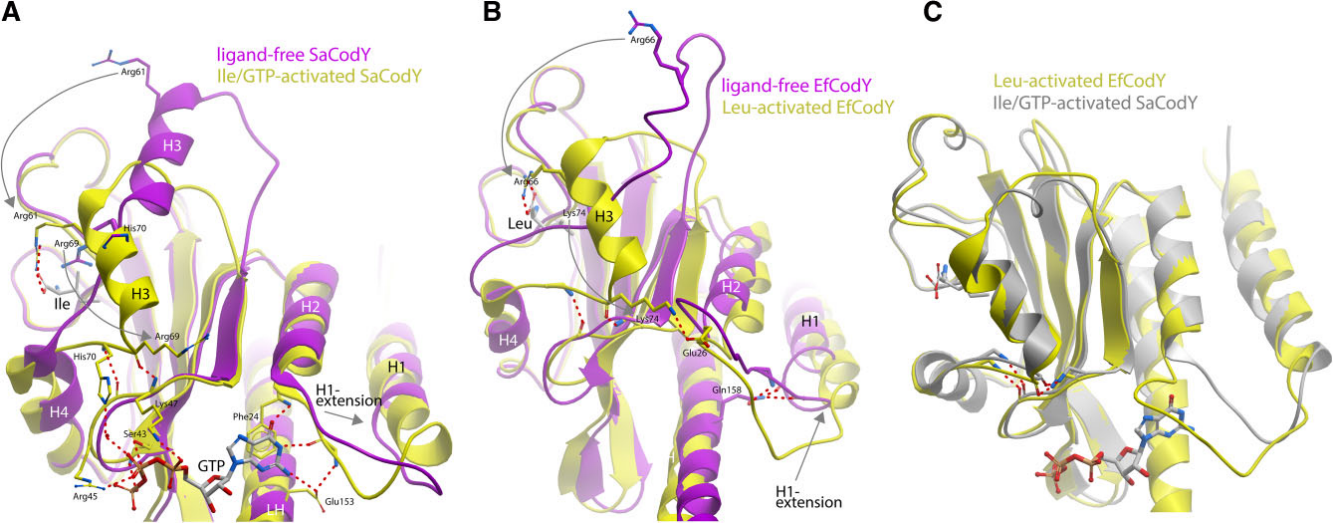
Ligand binding induces large conformational changes in the GAF domain. (**A**) Superimposition of ligand-free and ligand-activated SaCodY, highlighting the shift of helix H3. (**B**) Superimposition of ligand-free and ligand-activated EfCodY, highlighting the folding of the extended loop into helix H3 (**C**) Superimposition of the GAF domains of ligand-activated and DNA bound SaCodY (residues 25–155) and EfCodY (residues 30–160) shows the similarity of their structures.

To elucidate structural changes induced on the GAF domain by Ile and GTP, we compared the ligand-free and ligand-bound SaGAF domain structures. The most apparent structural change occurs on the outer face of the ß-sheet, in the region between ß-strands S2 and S3 including also helix H3. Here, Ile-binding induced a large shift of the entire helix H3 (residues Gln60–Glu68) (Figure 3A, Supplementary Figure S4). The ∼14 Å movement of the Cα atom of Arg61 in the ligand-bound structure exemplifies the magnitude of this shift from its position on the surface in the ligand-free structure (Figure 3A). In more detail, in absence of ligands, Arg69 in the H3–H4 linker, extends its side chain into the vacant Ile binding site making a hydrogen bond to the main chain carbonyl group of Val97. Following ligand binding and the H3-helix shift, Arg61 (located at the H3 N-terminus) occupies the equivalent position of Arg69. There it forms a salt bridge with the carboxylate group of the bound Ile molecule (Figure 3A). Meanwhile, Arg69, moves ∼11 Å towards the S1–S2 β-hairpin. Together with His70 and Ile71, it forms a ß-sheet interaction with Gly46, Lys47 and Ile48 in the S1–S2 loop. These two regions, the S1-S2 loop and the H3-H4 linker, compose the GTP-binding motifs 2 and 3 respectively (15). These in turn together form the binding pocket for the GTP phosphates. It appears that the induced ß-sheet interactions adjust residues Ser43, Arg44, Arg45, Lys47 in motif 2 to allow direct phosphate interactions and stabilize the large displacement of motif 3. This displacement positions His70 sufficiently close to form a water-mediated phosphate interaction.

The guanine moiety of GTP makes Watson–Crick-like interactions with the main-chain nitrogen atom of Phe24, the main chain oxygen atom of Val22, and the side chain carboxyl group of Glu153. Furthermore, the guanine base is stabilized by stacking on the side chain of Phe24. Residues Val22 and Phe24 reside in the H1-H2 loop region which composes the GTP binding motif 1 (15). In the ligand-free structure, elevated B-factors suggest that the H1-H2 loop region (residues Leu14-Phe24) has increased flexibility. The formation of the Watson–Crick-like interactions of the guanine base of GTP induces a conformational change that stabilizes the H1-H2 loop (Figure 3A). The loop backbone now hydrogen bonds with the side chain of linker helix-residue Glu153. Moreover, residues Leu14, Gln15 and Lys16 become α-helical, extending H1 with one turn and forming hydrogen bonds with residues in protomer B (e.g. to residue Thr148 in the linker helix, and to residues Gly118, Gly119 and Gly120 in the S3–S4 β-hairpin loop). Superimposition of monomeric ligand-free and dimeric-activated SaGAF domains shows that residues Gln15-Lys18 in the ligand-free domain cause steric clashes with the linker helix and S3–S4 loop of protomer B. This may explain the monomeric state of ligand-free SaCodY (Supplementary Figure S5).

In conclusion, our data suggest that: (i) Ile binding induces structural changes in the GTP-binding motifs 2 and 3, which enable the GTP phosphate interactions necessary for efficient GTP binding and (ii) the guanine base of the bound GTP nucleotide induces the structural changes in motif 1 that are then propagated to the protomer-protomer interface. This implies that Ile and GTP binding are structurally linked and that Ile and GTP act synergistically. The affinity of SaCodY for the *hutP* operator sequence showed that efficient DNA binding activity is indeed dependent on the presence of both ligands (Figure 1).

### Activated SaCodY and EfCodY are structurally similar

We determined the crystal structure of the ligand-free form of EfCodY; however, we did not manage to obtain diffracting crystals of Leu-bound EfCodY. Fortunately, the crystal structure of the EfCodY-DNA complex—presented in more detail below—had Leu-bound GAF domains. The crystal structure of the SaCodY–DNA complex—also presented below—had Ile/GTP-bound GAF domains. The Ile/GTP-bound GAF domains of SaCodY in its free and DNA-bound forms are essentially identical, demonstrating that the GAF domains do not undergo a structural change upon DBD domain-DNA interaction. This allowed us to characterize Leu-activation of EfCodY by analysing the GAF domains in the ligand-free and the Leu/DNA-bound EfCodY and to compare Leu-activation of EfCodY to Ile/GTP-activation of SaCodY.

**Figure 4.**
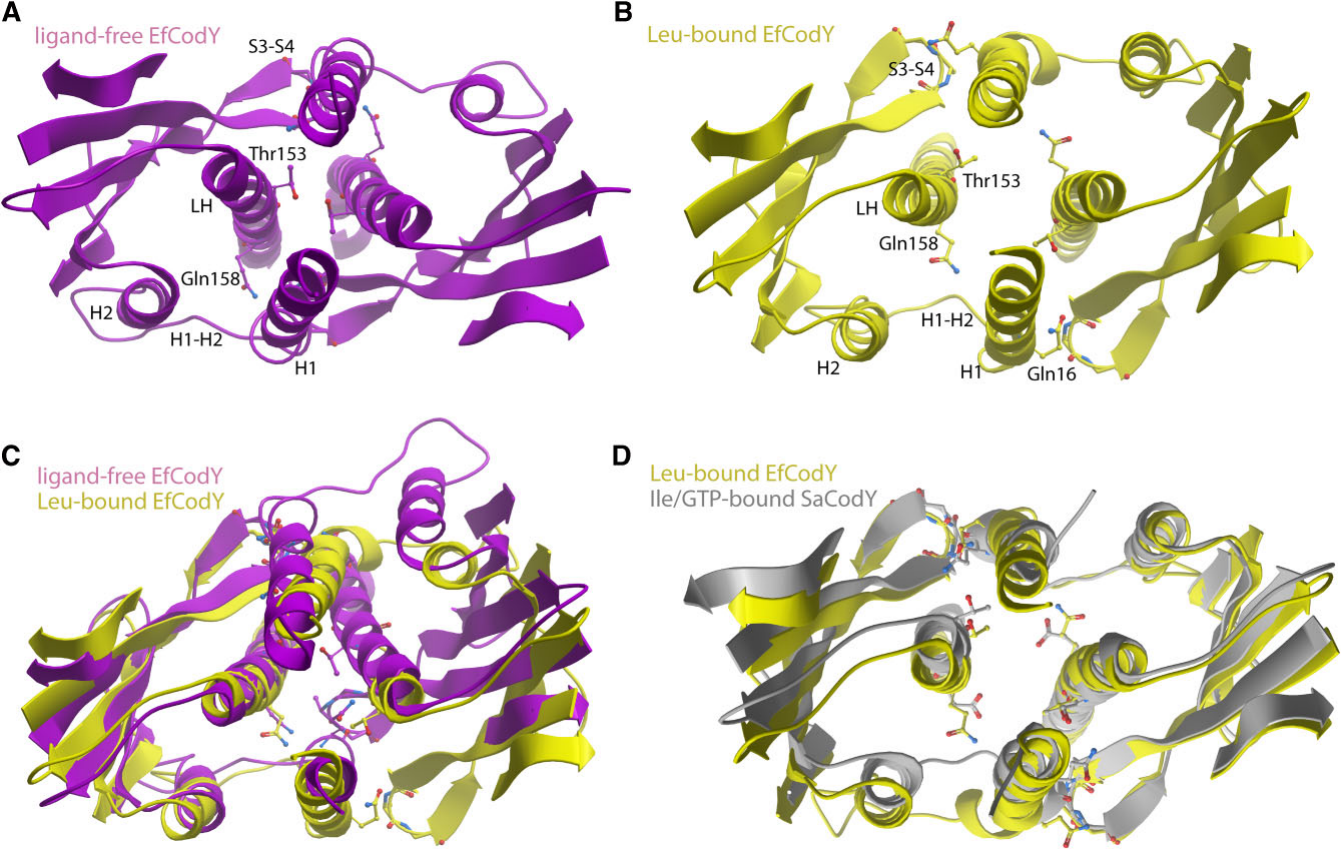
Leu binding induces a major rearrangement of the 4-helix bundle at the GAF domain protomer-protomer interface. Top view ribbon representations of the GAF domain dimers in (**A**) ligand-free EfCodY and (**B**) Leu-bound EfCodY. In (**C**), the ligand-free (purple) and Leu-bound (yellow) dimers are superimposed highlighting the major rearrangement of the 4-helix bundle. In (**D**), the ligand-free dimer (yellow) is superimposed on the dimer in Ile/GTP-bound SaCodY (gray) highlighting a very similar 4-helix bundle arrangement. The overlay was generated by superimposition of residues 95–140 in EfCodY (90–135 in SaCodY). For clarity, the two long loops on the outer face of the β-sheet are excluded.

The structures of the activated GAF domains in Leu-bound EfCodY and Ile/GTP-bound SaCodY are remarkably similar. However, there are noticeable differences in the structural changes leading to the activated GAF domains. In ligand-free EfCodY, residues Leu60-Gln78/Glu55-Ser73 (EfCodY/SaCodY sequence numbering = shift –5) form an extended loop with a few loop residues involved in crystal contacts. The fold of the loop in all four protomers is identical even though the crystal contacts are not identical, suggesting that the loop structure in the crystal structure also represents the structure in solution. The most apparent difference of the loop structure to that in SaCodY is the absence of helix H3 (Supplementary Figure S6). Nevertheless, Lys74 (Arg69 in SaCodY) extends into the vacant Leu binding pocket. Leu binding induces the H3-formation associated with a large displacement of loop residues Lys74, Lys75 and Phe76. These residues now reside in the H3-H4 linker forming a ß-sheet interaction with Gly51, Asp52 and Leu53 in the S1–S2 loop—similar to what we observed in SaCodY (Figure 3B). Arg66 moved into the position of Lys74 and occupies the equivalent position to Arg61 in Ile/GTP-activated SaCodY, forming a salt bridge with the carboxylate group of the bound Leu-molecule. The H1–H2 loop, which in EfCodY contains an insertion of the four additional residues 25-AlaGluLeuPro-28, also changes its conformation upon Leu-binding including breaking of the hydrogen bond between the loop backbone and Gln158 in the linker helix (the homologue of Glu153 in SaCodY). Moreover, similar to what we observed in SaCodY, residues Gln16, Lys17 and Asn18 become α-helical. These residues are in contact with the S3-S4 loop and linker helix of protomer B in both the ligand-free and Leu-bound EfCodY dimer. The H1-extension therefore triggers a concerted movement of the S3-S4 loop and linker helix, leading to a striking different 4-helix bundle arrangement at the GAF dimer interface (Figure 4). The 4-helix bundle arrangement in activated SaCodY and activated EfCodY, however, is very similar to each other. The corresponding 131 Cα atoms of the GAF domain in DNA-bound SaCodY and EfCodY can be superimposed with root-mean-square deviations (RMSD) of 0.8 Å (Figures 3C, 4D).

Importantly, Lys74 in the H3–H4 linker, stabilized by the ß-sheet, now moved close enough to the H1–H2 loop to form a direct salt bridge with the insertion residue Glu26. In EfCodY, the structural changes in the BCAA-binding site are thus directly coupled to changes in the H1–H2 loop—as opposed to via GTP in SaCodY. Consistent with this notion, DNA binding in EfCodY can be activated by Leu alone (Figure 1). To further confirm the importance of Lys74 for EfCodY activation, we analysed a Lys74Ala mutant. Indeed, the mutant binds Leu with comparable affinity to the wild type but has greatly reduced DNA-binding activity (Supplementary Figure S7). This argues for a crucial role of Lys74 in propagating the structural changes in the Leu-binding site to the H1–H2 loop and protomer-protomer interface.

### SaCodY and EfCodY in complex with DNA

**Figure 5.**
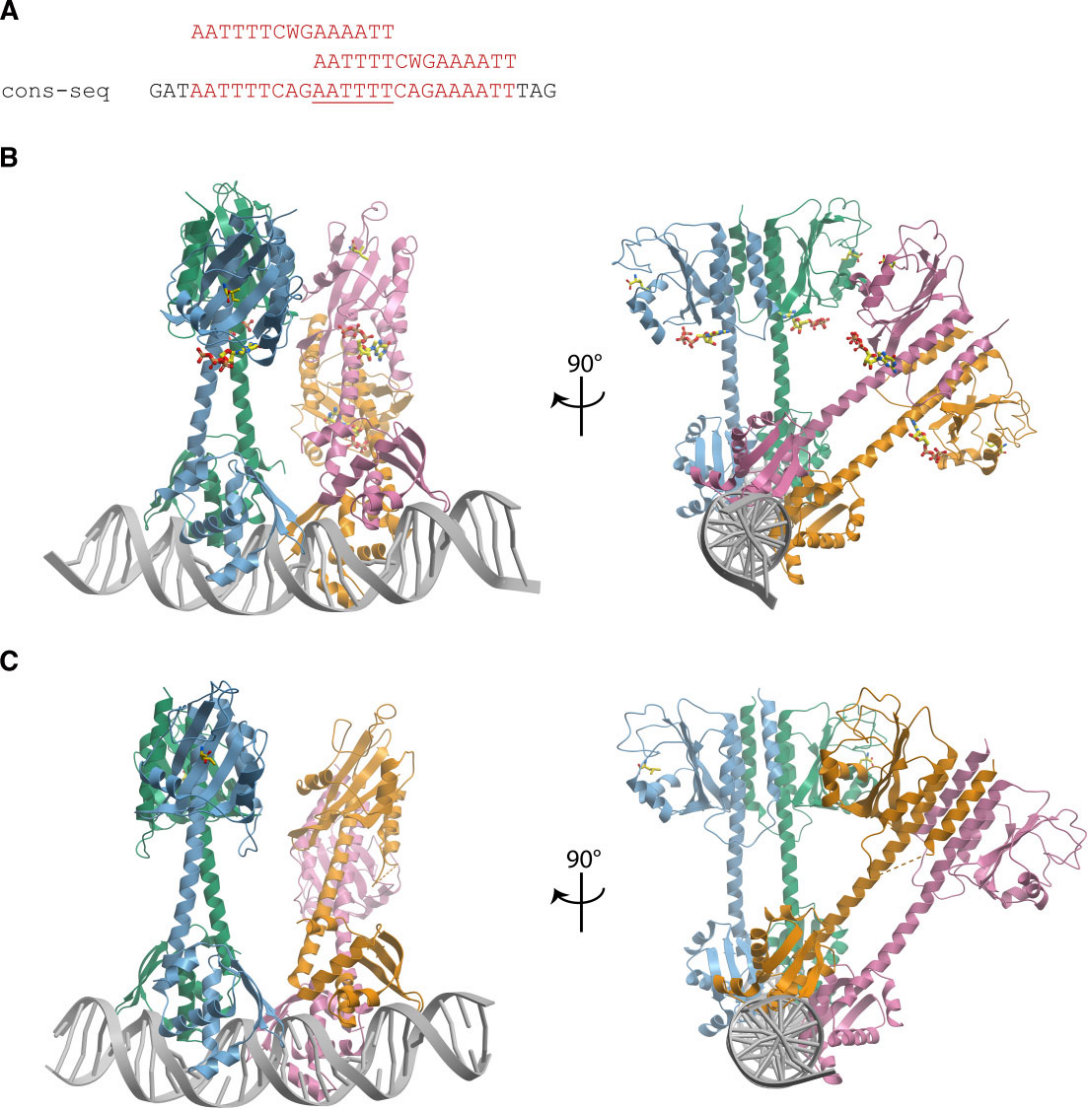
Overall structure of the CodY-DNA complexes. (**A**) The DNA used for crystallization contains two copies of the 15-nt binding site with a 6-nt overlap (underlined). The consensus sequence is shown on top. The 3′-binding site matches the consensus sequence, the 5′-binding site contains two mismatches to the consensus sequence and lacks the pseudo-palindromic character. (**B**) The biological unit of the SaCodY-DNA complex. (**C**) EfCodY-DNA complex.

To understand the structural basis of DNA recognition and cooperativity in CodY, we determined crystal structures of SaCodY and EfCodY in complex with DNA. To create high-affinity CodY binding sites for this purpose, we designed DNAs that contained overlapping binding sites with optimized sequence similarity to the consensus sequence (Figure 5A). We obtained crystals of a 30-bp DNA bound to SaCodY and EfCodY and determined the structures at a resolution of 3–3.2 Å. In the crystals, two CodY dimers bind to a single DNA duplex. The SaCodY-DNA complex was co-crystallized with Ile and GTP (Figure 5B). Both ligands are clearly visible in the electron density of the four independent protomers. The EfCodY complex was co-crystallized in the presence of Leu, yet Leu bound only to protomers A and B (dimer 1) (Figure 5C, Supplementary Figure S2). In protomers C and D (dimer 2), the residues Glu58-Lys75 involved in the H3-helix shift had overall weaker electron density compared to ligand-free EfCodY. Furthermore, the H1-H2 loop defined by residues Asn19-Glu26 had weaker electron density in all four protomers.

### Description of CodY dimers bound to DNA

**Figure 6.**
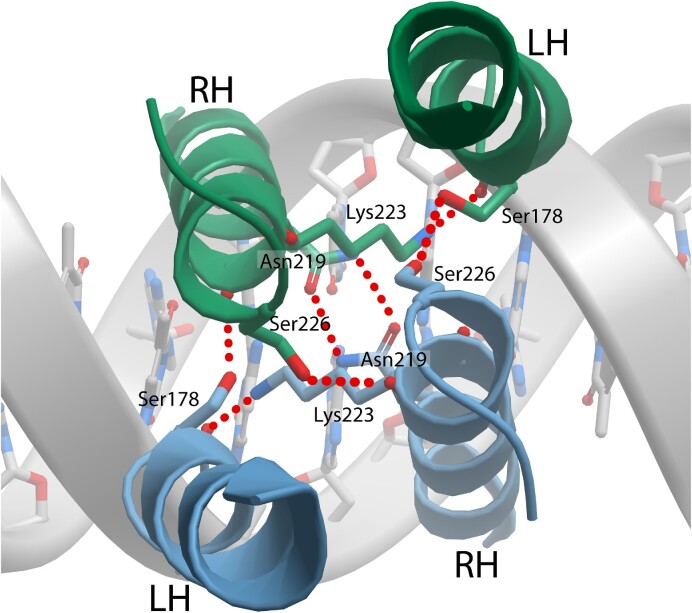
The recognition helices dimerize upon DNA-binding. Top view ribbon representation of the linker helix-recognition helix protomer–protomer interface. Protomer A is colored in blue, protomer B in green. Hydrogen bonds stabilizing the interface are indicated. LH indicates linker helix and RH recognition helix.

In the CodY-DNA complex, the SaCodY- and EfCodY-dimers adopt a dumbbell-shaped structure in which the dimerized GAF and DBD domains form two physically separated lobes connected by the extended linker helices (Figure 5). The DNA-bound CodY-dimer is stabilized by polar and hydrophobic interactions between the CodY-protomers, comprising residues in both the GAF and DBD lobes as well as the linker helices. The dimer interface buries ∼1900 Å^2^ of solvent-accessible surface area, with the GAF lobe accounting for ∼67% of the interface and the DBD lobe for ∼22%. In the GAF lobe, H1 and the linker helix form the 4-helix bundle with a mainly hydrophobic protomer-protomer interface. In the DBD lobe, the C-terminal ends of the recognition helix are wedged in between the C-terminal ends of the linker helices. Mainly polar interactions are formed between recognition helix residues from both protomers—assembling an unusual recognition helix dimer—and between recognition helix residues of one protomer with linker helix residues of the other protomer (Figure 6). Residues in the entire CodY protomer-protomer interface are highly conserved—most notably in the DBD domains where the recognition helix interface-residues are invariant in all CodY proteins (Supplementary Figure S8).

### Spatial arrangement of CodY dimers bound to DNA

**Figure 7.**
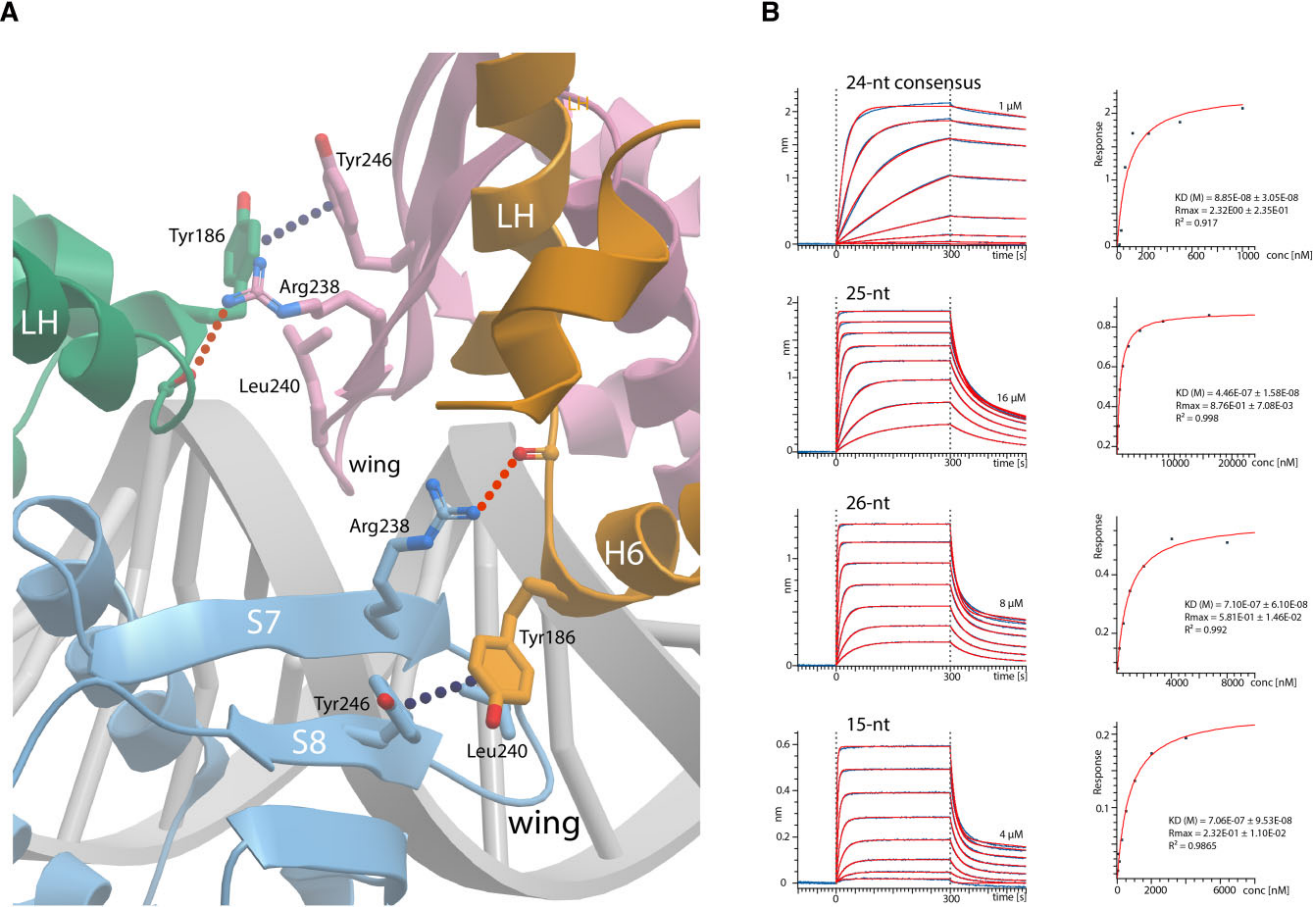
Cross-dimer interactions are required for the cooperative binding of CodY to DNA. (**A**) Ribbon representation of the cross-dimer interaction site mediated by S7-S8 hairpin and H6 N-terminus in the central shared half-site of the overlapping biding sites. Interacting residues are depicted in stick representation, hydrogen bonds are indicated red and π-stacking interactions in dark blue. EfCodY-protomer A is blue, B green, C orange and D purple. (**B**) To disrupt cross-dimer interactions we inserted one and two A–T base pairs into the central half-site creating 25-nt and 26-nt overlapping binding sites. Shown are representative bio-layer interferometry sensorgrams of a twofold dilution series of EfCodY binding to the 24-nt sequence optimized overlapping sites, the 25-nt and 26-nt sites, and the single 15-nt consensus site. The highest concentration of the series is indicated. The curve fittings are depicted in red. The affinity plot response vs. SaCodY concentration—derived from the curve fittings—are shown to the right. EfCodY has reduced affinity for the 25-nt and 26-nt sites, with binding cooperativity completely lost for the 26-nt sites, for which EfCodY has the same affinity as for the single site (710 and 706 nM). LH indicates linker helix.

A prominent feature of the DNA complex structure is the relative spatial arrangement of the two CodY-dimers. The dimers are related by a 60° rotation and a 30-Å translation along the DNA, binding one side of the DNA helix. The spatial relationship of the two dimers of SaCodY is defined by a crystallographic axis. In the *Ef*-complex, the DBD domains of the two dimers are related by a 60° rotation as in the *Sa*-complex; however, their GAF domains are related by a ∼45° rotation due to a shift in the position of the N-terminal parts of the linker helices (Figure 5). This dimer arrangement is stabilised not only by interactions of each dimer with the DNA, but also by direct interactions between the two dimers, referred to as cross-dimer interactions. A cross-dimer interaction is formed at the DBD domains where the S7-S8 β-hairpin of one dimer and the H6 N-terminus of the other dimer reciprocally interact with each other, covering an interface of ∼380 Å of the solvent-accessible surface area. The interface is rigid and essentially the same for the *Sa*- and *Ef*-complexes. Interactions are mediated by residues Arg238, Leu240, and Phe246 (S7-S8 ß-hairpin) and 184-Leu,Ser,Tyr,Ser-187 (H6 N-terminus, EfCodY numbering) (Figure 7A). These residues are strictly conserved in all CodY-proteins except for Tyr246 that is also found as Phe or His. As noted above, we found no experimental evidence (by either mass photometry or SEC-MALS) that SaCodY or EfCodY forms tetramers in the absence of DNA. This argues that the stable cross-dimer interaction seen in the crystal is DNA-dependent.

### Cross-dimer interactions are required for cooperative DNA-binding

To assess the functional relevance of the DBD–DBD cross-dimer interaction for cooperativity, we replaced residues Tyr186, Arg238, Leu240 and Tyr246 in EfCodY with Ala and evaluated the quadruple mutant for DNA affinity. The EfCodY-mutant is still dimeric in solution (data not shown from mass photometry and SEC-MALS); however, bio-layer interferometry showed that this mutant was unable to bind to the 24-nt sequence-optimized overlapping sites (Supplementary Figure S9). This failure to bind DNA could be because the mutated residues might not only mediate cross-dimer interactions, but also promote DNA-binding, a notion supported by close proximity of mutated residues and protein–DNA interface. Therefore, we used a different approach and reduced the binding site overlap from 6-nt to 5-nt and 4-nt. We expected that the altered angle and increased distance between the two dimers would prevent cross-dimer interactions. Affinity measurements showed that EfCodY binds sequence-optimized 25-nt and 26-nt overlapping sites with low affinities, comparable with that to a single site (Figure 7B). This indicates that lateral stabilization of CodY-dimers on DNA through cross-dimer interactions and tetramerization is required for the cooperative binding of CodY to DNA.

### Non-canonical DNA binding by CodY

**Figure 8.**
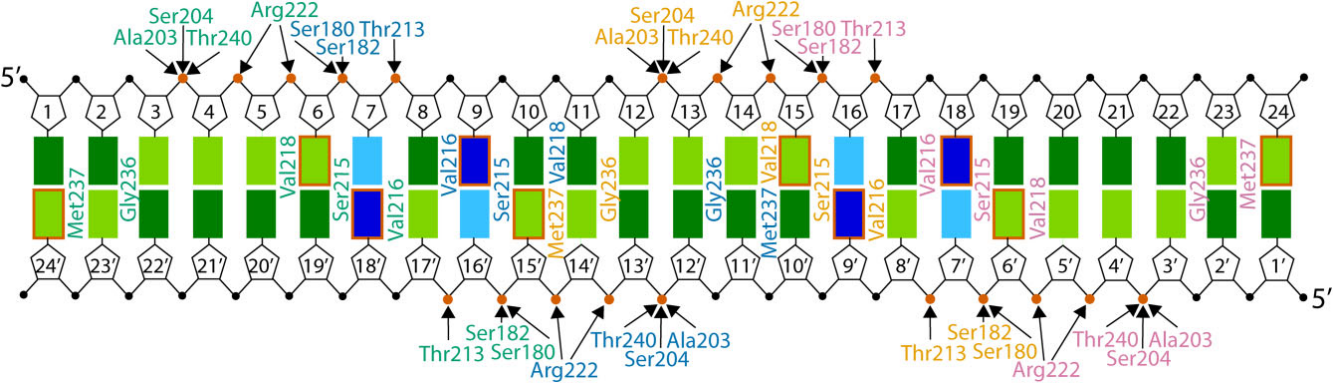
CodY-DNA recognition. Schematic diagram summarizing the contacts between CodY and the 24-nt overlapping binding sites. Rectangles represent DNA bases: T (light green), A (dark green), C (light blue), and G (dark blue). CodY residues of protomer A are labeled blue, protomer B green, protomer C orange, and protomer D purple. Arrows indicate hydrogen bonds. Red circles represent phosphate groups hydrogen bonded with CodY-residues. Bases with a red border represent bases hydrogen bonded with CodY-residues. The non-template strand nt are indicated by a prime sign. The three base pairs at the 5′- and 3′-end respectively are excluded.

The two symmetrically positioned wHTH motifs in each CodY dimer bind to the 15-nt binding site such that each motif recognizes one half-site. The dimerised recognition helices sharply angle away from the DNA axis and insert the N-terminal ends centrally into the major groove. The S7–S8 ß-hairpins extend to each side and insert the wings into the adjacent minor grooves—at the binding site 5′- and 3′-ends. Consequently, in the DNA containing the 24-nt overlapping sites, the two wings in the central shared half-site (nt 9–16) insert into the minor grooves next to the centre of each other's site. Note that—although CodY contains a canonical wHTH-motif (36)—CodY-wHTH-DNA binding is non-canonical in that dimerized recognition helices insert into a single major groove. This is in contrast to canonical wHTH binding, in which separate recognition helices insert singly into consecutive major grooves (Supplementary Figure S10). An recognition helix-dimerized form of the wHTH-motif bound to DNA similar to that of CodY has been reported for FadR (37) and TubR (38). The DNA interacting residues are conserved in all CodY proteins, and SaCodY and EfCodY form essentially identical interactions with the DNA (superimposing DBD-residues 175–245 in SaCodY with the homologous residues 180–250 in EfCodY gives RMSD values of between 0.4–0.5 Å). The two CodY-dimers interact with all 24 nucleotide pairs of the overlapping binding sites burying ∼3500 Å of solvent-accessible surface area. DNA interactions are primarily mediated by hydrogen bonds of residues with the sugar–phosphate backbone of the DNA, indicative of an indirect or shape readout mechanism. As will be discussed, only two base-specific hydrogen bonds, namely with residues Ser215 and Met237 (numbering from here on corresponds to SaCodY), are formed between each protomer and the half-site DNA (see Figure 8 for a schematic diagram).

### Interactions between DBD domain and DNA

**Figure 9.**
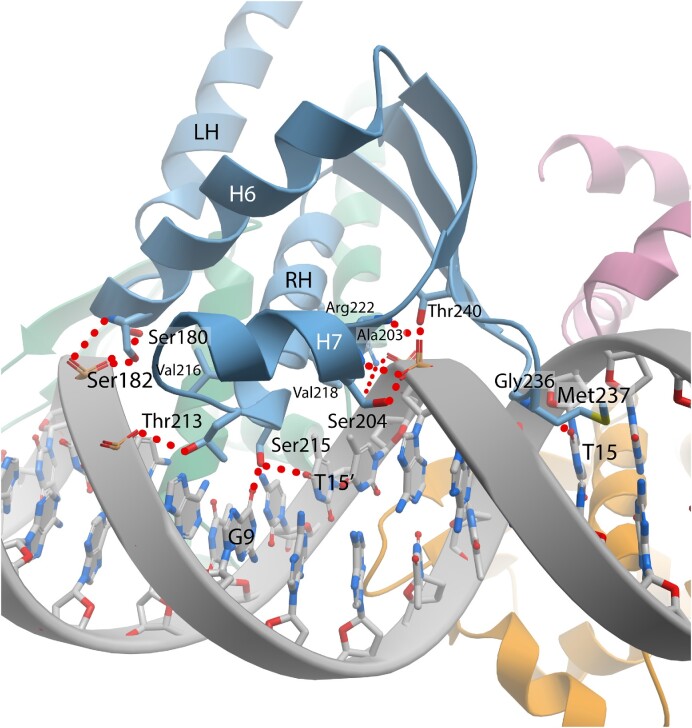
CodY-DNA recognition. Ribbon representation of SaCodY protomer A (blue) interacting with major and minor grooves of the consensus binding site. Interacting residues are depicted in stick representation. Hydrogen bonds between residues and DNA backbone as well as between Ser215 and Met237 and DNA bases in major and minor groove are indicated. LH indicates linker helix and RH recognition helix. For clarity, the ribbon representation for residues 244–260 is excluded.

What follows is a detailed description of the interactions between protomer B of SaCodY and the 5′ half-site DNA (Figure 9). The position of the recognition helix in the major groove is stabilized by hydrogen bonds of recognition helix residues Thr213 and Arg222 to backbone phosphates of T^5^, T^6^ and T^17’^ located on opposite sides of the major groove (the prime-sign indicates template strand nt). The recognition helix position allows the side chain of Val216 and Val218 to make hydrophobic contacts with the methyl group C7 of the thymine bases T^6^ and T^17’^. Ser215, located at the recognition helix N-terminus, forms the only base-specific hydrogen bond between the recognition helix and major groove bases. The side chain hydroxyl group projects towards the DNA major groove floor and forms a hydrogen bond with the carbonyl oxygen atom O4 of T^6^ or O6 of G^18’^. Absence of electron density for Ser215 side chains indicate that the side chain can adopt different rotamer conformations that allow switching between alternative hydrogen bonds with bases T^6^ or G^18’^. Interestingly, the rotamers potentially allow for hydrogen bonds also with bases G^6^ and T^18’^, and weaker hydrogen bonds with G^7^ and T^7^. Therefore, in addition to the consensus T^6^C^7^/G^18’^A^19’^ base-pair step, T^6^N^7^/N^18’^A^19’^, N^6^C^7^/G^18’^N^19’^, G^6^N^7^/N^18’^C^19’^, N^6^A^7^/T^18’^N^19’^ and (N^6^G^7^/C^18’^N^19’^, N^6^T^7^/A^18’^N^19’^) base-pair steps could be accommodated without significant loss of binding specificity (N represents any base; base pairs steps in parentheses indicate weak hydrogen bonds with Ser215). In agreement, the C^7^–G^18’^ base pair is frequently missing from actual CodY-binding sites but its absence does not prevent efficient regulation of the corresponding genes by CodY (39).

Two α-helix dipole interactions with the DNA backbone further stabilize the HTH-unit in the major groove (Figure 9). H7 packs its partial, positively charged N-terminus against the backbone phosphate at position T^4^. The T^4^ phosphate is deeply buried in a cleft formed by the H7 N-terminus and the S7-S8 β-hairpin located in major and minor grooves, respectively, and is hydrogen bonded to the main chain N-cap nitrogen atoms of Ala203 and Ser204 (H7 N-terminus), and the hydroxyl group of Thr240 (S8). On the opposite side of the major groove, H6 packs its N-terminus against the DNA backbone phosphate at position C^16’^. The C^16’^ phosphate is hydrogen bonded with the main-chain N-cap nitrogen atom of Ser182 as well as the side-chain hydroxyl groups of Ser180 and Ser182. These residues reside in the invariant 179-Leu, Ser, Tyr, Ser-182 sequence, mediating the cross-dimer interactions. In the shared half-site, the C^16^/C^16’^ backbones are wedged between the H6 N-terminus and S7-S8 β-hairpin of the two dimers. This suggests that the H6-DNA contacts may play a role in proper positioning of Tyr181 for cross-dimer stacking interactions with Phe241 in β-strand S8 (Tyr246 in EfCodY) (Figure 7A).

The wing element of the wHTH motif is formed by the short turn between β-strands S7 and S8 comprising residues Leu235, Gly236, and Met237. The wing penetrates deeply into the AT-minor groove at position T^1^-A^3^/ T^24’^-A^22’^, and—in the shared half-site—is positioned between the H6- and H7-N-termini of the two dimers. The embedded main chain of Gly236, as well as main and side chains of Met237, extend over the shape of the minor groove floor. Note that AT minor grooves are usually recognized by positively charged Arg residues (or less frequently Lys residues) to complement the local shape and enhanced negative electrostatic potential (40). Met237 also forms one base-specific hydrogen bond with its main chain nitrogen atom to the carbonyl oxygen atom O2 of T^24’^ (Figure 9). Thus, in the shared half-site, the T^15^ and T^15’^ bases hydrogen bond with both dimers; the minor groove base edge interacts with Met237 from one dimer and the major groove base edge interacts with Ser215 from the other. In agreement, sequence logos of the 24-nt overlapping sites from different species show that T^15^ and T^15’^ are almost completely conserved (20,21). In line with the structural and base frequency data, we found that mutation of the T to A or G strongly reduces DNA binding of SaCodY and EfCodY (Supplementary Figure S11).

### Binding of CodY locally distorts the double helix

**Figure 10.**
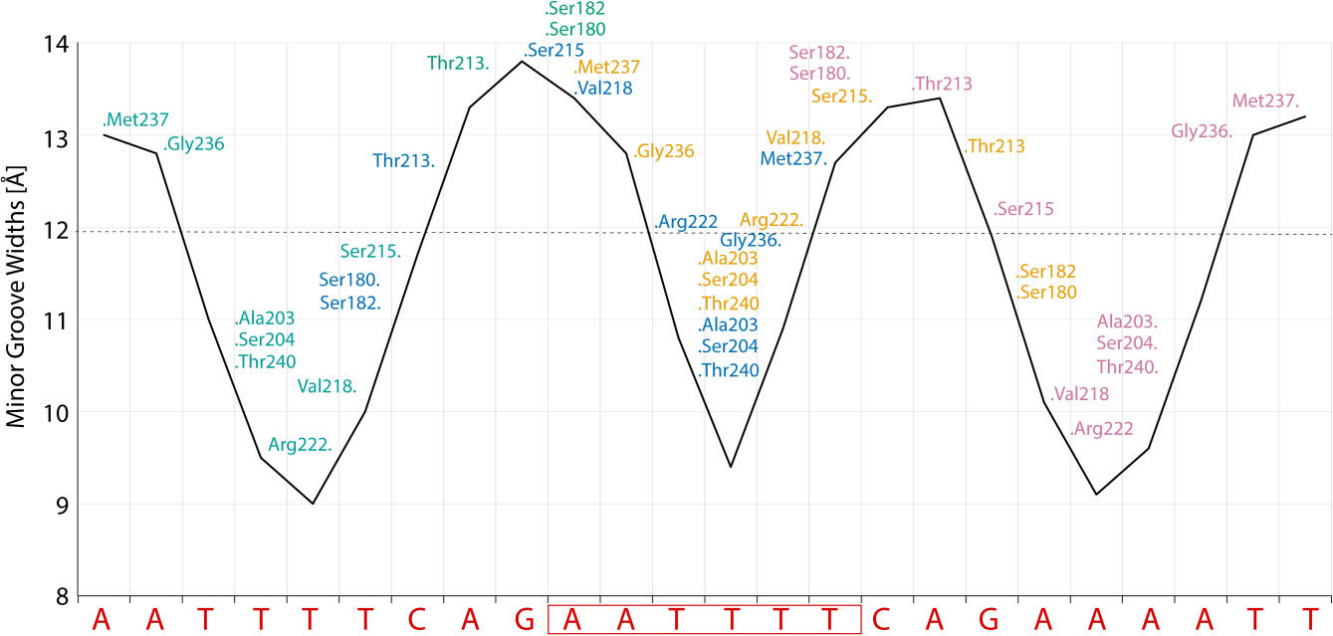
The DNA in the *Sa-* and *Ef*-complex shows a strong oscillation of minor groove widths. Minor groove widths are plotted over the length of the SaCodY-bound DNA. The y-axis values represent inter-strand phosphate-phosphate distances. The dashed line represents the canonical minor groove width for B-DNA. CodY residues are plotted approximately where they interact along the DNA sequence. The sequence of the overlapping sites is shown along the x-axis with the shared half-site boxed. Note that the minor groove compression for the shared half-site is less severe. Changes in major groove widths are much less pronounced (not shown).

Globally, the DNA in the complex adopts a slightly bent B-form structure; however, locally, the DNA deviates from the canonical B-form. Strikingly, the minor grooves along the three AT-sequences (position A^1^-T^6^, A^10^-T^14^, A^19^-T^24^) are unusually narrow. Along three base pairs of the two peripheral AT-sequences (T^3^-T^5^, T^20^-T^22^), the minor groove widths average 9.5–9.6 Å (P-P distance; compared to 12.0 Å in the canonical B-form) with a minimum of 9.0 Å. The minor groove along the two CAG sequences is moderately widened (maximum 13.8 Å), causing a strong oscillation in minor groove widths (Figure 10). Notably, the minor groove compression for the central AT-sequence—bound by two wings—is less severe (average 10.4 Å, minimum 9.4 Å), indicating that the wing insertion widens an intrinsically narrow minor groove. An important feature of CodY target sites thus appears to be the narrow shape and deformability of the minor grooves.

### CodY activation: communication between ligand bound GAF domains and DBD domains

**Figure 11.**
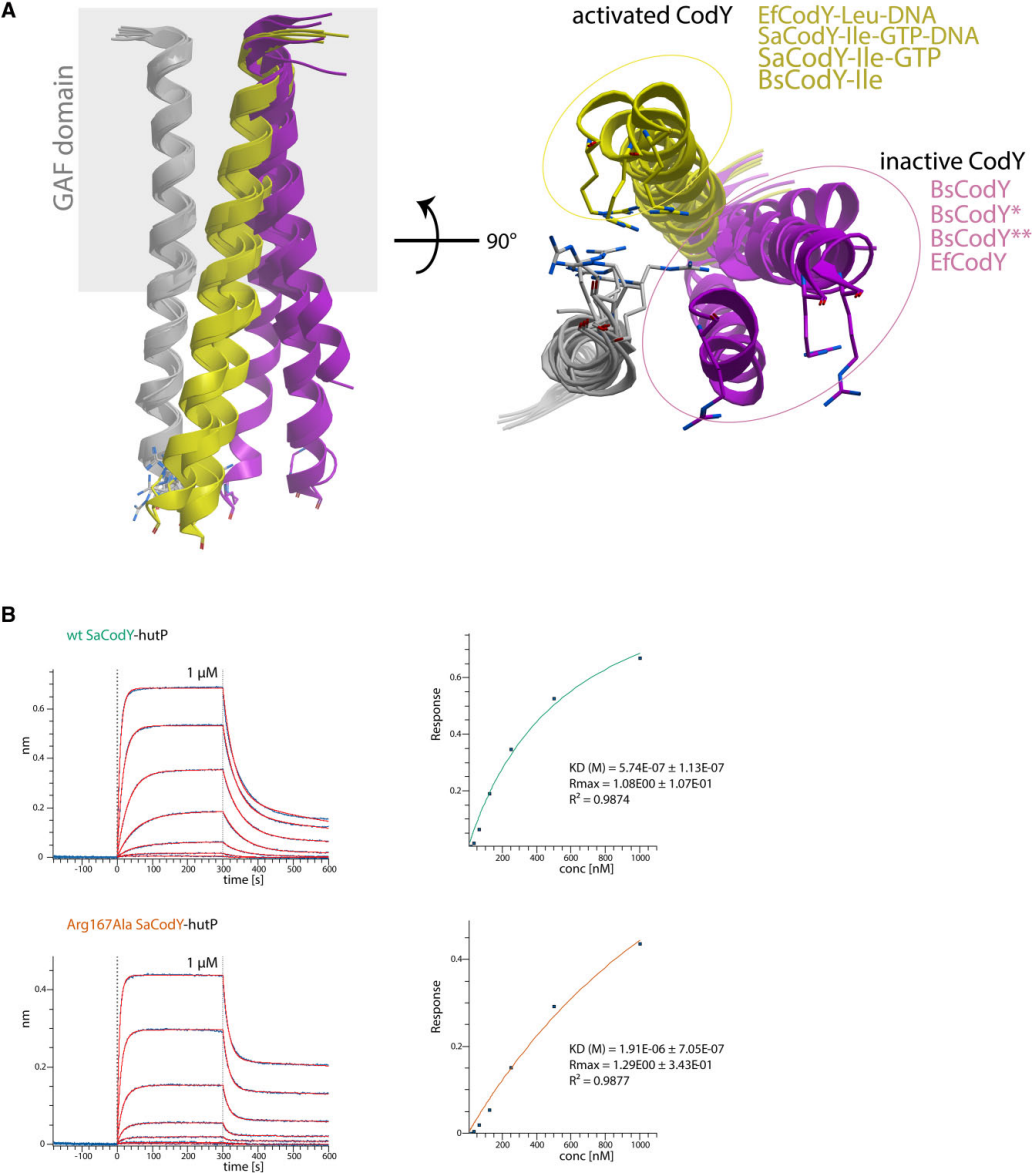
The relative orientations of the linker-helices differ between inactive and activated CodY. (**A**) Superimposition of linker helices from eight CodY-structures. The ribbon structures for the superimposed linker helices are depicted in gray (residues 135–167 for SaCodY and BsCodY, and 140–172 for EfCodY). The ribbon structures for the other linker helix in the dimer are depicted in yellow (ligand-bound structures) and purple (ligand-free structures), which highlights that the relative orientations of the linker helices in the ligand-activated CodY structures are very similar and differ from those in inactive CodY. The gray square indicates the GAF domain suggesting that the different linker helix orientations originate from packing differences within the GAF domain. EfCodY-Leu-DNA (chain A and B, this work), SaCodY-Ile-GTP-DNA (chain A and B, this work), SaCodY-Ile-GTP (chain A and B, PDB code 5ey0), BsCodY-Ile (chain A and B, PDB code 2b18), BsCodY (chain A and B, PDB code 2gx5), BsCodY* (crystal form B, chain A and H, PDB code 5nlh), BsCodY** (crystal form C, chain A and B, PDB code 5loj) and EfCodY (chain A and B, this work). The invariant Arg167 is shown as stick representation and highlights their stacking interaction in activated CodY. (**B**) Representative bio-layer interferometry sensorgrams of a twofold dilution series of wild-type SaCodY and an Arg167Ala mutant SaCodY interacting with the overlapping binding sites in the *hutP* operator of *Bs*. The highest concentration of the series is indicated. The curve fittings are depicted in red. The affinity plot response vs. SaCodY concentration—derived from the curve fittings—shows a significant decrease in DNA-affinity for the Arg167Ala variant.

Because the GAF domains are physically separated from the DBD domains, ligand-binding must be communicated via the linker helices. When comparing the available CodY structures, we observed different linker helix orientations that allowed some positional flexibility of the DBD domains, by adopting different orientations relative to one another and to the GAF domains. Moreover, our structural comparison suggested that the different linker helix orientations originate from linker helix packing differences within the GAF domains. Our data indicate that—within the GAF domain—ligand-induced conformational changes are propagated via the H1–H2 loop to H1 and the N-terminal part of the linker helices. This leads to a different packing of the linker helix in the 4-helix-bundle that forms the protomer-protomer interface (Figure 4). In agreement, superimposition of the linker helix structures shows that the linker helix orientations in activated CodY (BsCodY (18,35), SaCodY (15), and DNA-bound SaCodY and EfCodY presented here) significantly differ from those in inactive CodY (BsCodY (18,41), and EfCodY presented here) (Figure 11A). Notably, in the orientation of activated CodY, the invariant Arg167 from both linker helices forms stacking interactions with each other. This is not seen in inactive CodY. We speculate that the stacking interactions of this Arg167 facilitate the recognition helices to wedge in between the linker helices and to form the active protomer-protomer interface. In agreement, we found that an Arg167Ala mutant had strongly reduced DNA affinity (Figure 11B). However, CodY-DNA recognition is more than a simple docking of preformed DBD domains onto the DNA. This is shown by the activated free SaCodY structure (15), in which the symmetric DBD-DBD interface (including the recognition helix dimer) is not yet formed. Moreover, the DBD domains in the activated free form retain significant flexibility, showing that the DBD-dimer folds into a highly-ordered lobe only upon DNA binding.

## DISCUSSION

*Sa* and *Ef* are opportunistic pathogens that pose a major risk to human health, a risk increased by the rising prevalence of antimicrobial resistant *Sa* and *Ef* strains. Hence, there is an urgent need to better understand the mechanism by which these bacteria switch from their commensal to pathogenic state. A growing body of evidence suggests that CodY plays a critical role in disease development by reorganizing metabolism and activating virulence gene expression (2–4).

**Figure 12.**
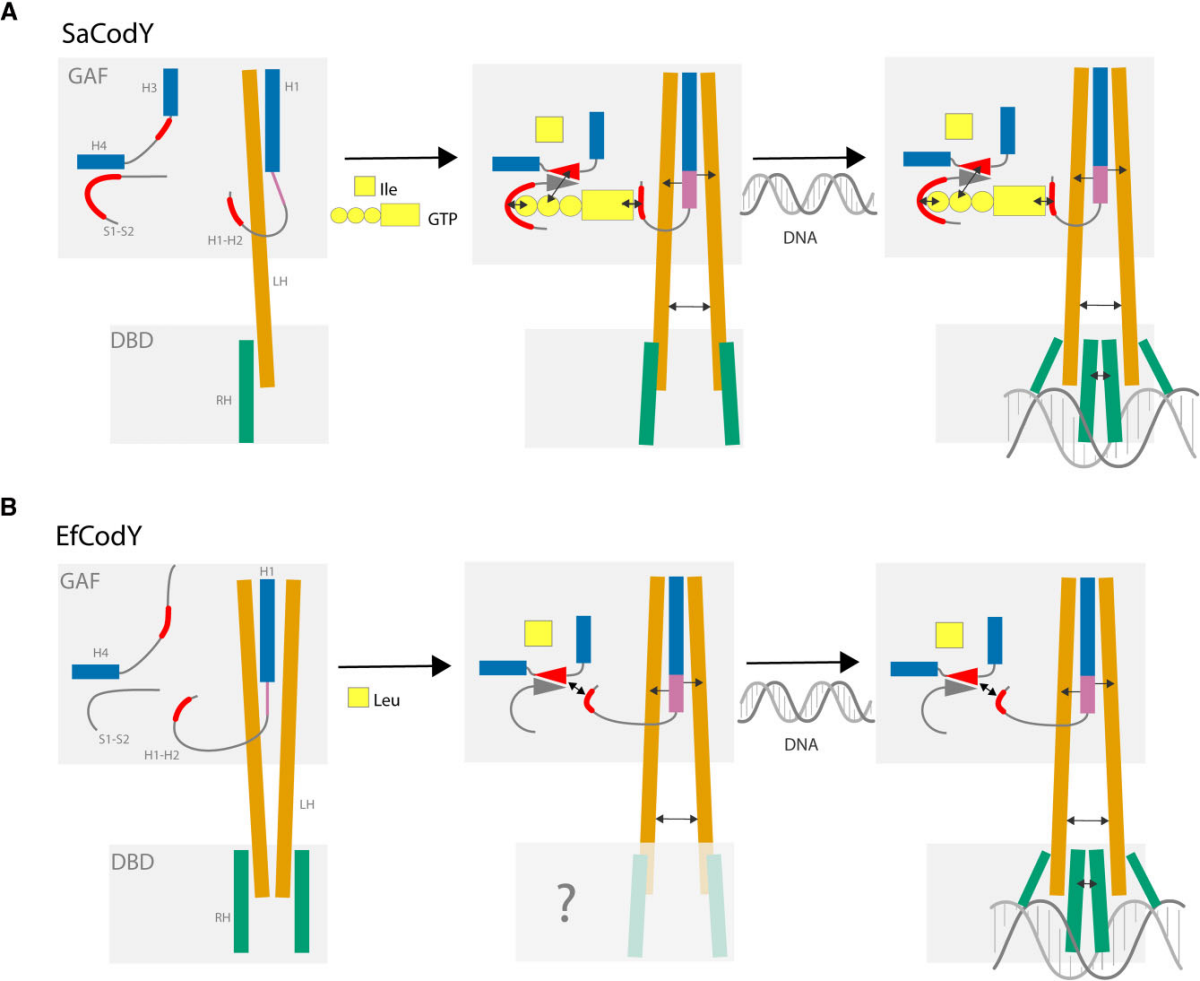
Schematic illustration of DNA-binding activation of SaCodY and EfCodY. (**A**) In SaCodY, Ile-binding triggers a movement of H3 that brings the H3–H4 linker and S1–S2 loop together (β-strand β-strand formation) and allows GTP binding. When bound, the guanine base of GTP interacts with the H1–H2 loop, which causes a C-terminal extension of H1 with 1–2 turns. At this stage, the DBD domains are less flexible than in their ligand-free form but primed for DNA binding. (**B**) In EfCodY, Leu binding triggers formation of H3 that brings the H3–H4 linker and S1–S2 loop together (β-strand β-strand formation). The β-sheet in turn interacts with the four-residues extended H1–H2 loop, which causes a C-terminal extension of H1 with 1–2 turns. As for the DBD domains in activated SaCodY shown in (A), we expect the DBD domains in activated EfCodY to be primed for DNA binding. Arrows indicate interactions.

The mechanism by which CodY-DNA binding is activated can differ for CodYs from different bacterial genera. Differences include the GTP responsiveness, the nature of the BCAA activator, and the oligomeric state of the ligand-free form. Mechanistically, it is proposed that ligand binding causes a reorientation of the linker helices and thus primes the DBD domains for DNA binding (15,18). Exactly how ligand binding leads to changes in linker helix orientation, however, remained puzzling. Our biochemical and structural data of ligand-free and ligand-bound forms of SaCodY and EfCodY now allow for a more detailed understanding of BCAA/GTP signalling and, interestingly, reveal mechanistic differences and similarities (Figure 12).

In both SaCodY and EfCodY, ligand-binding induces a large rearrangement at the BCAA-binding site, causing critical residues to move and form β-sheet interactions with S1-S2 loop residues. Furthermore, for both SaCodY and EfCodY, ligand binding causes a conformational change of the H1–H2 loop associated with a C-terminal extension of H1 with 1–2 turns; this conformational change has also been observed in BsCodY (35). Conformational changes at the BCAA binding site are thus propagated to the protomer-protomer interface composed of the 4-helix bundle of H1 and linker helices that defines the relative linker helix orientations. In SaCodY, the conformational changes at the Ile binding site are propagated to the H1–H2 loop via GTP, explaining why Ile and GTP activate DNA-binding synergistically—an observation also reported for BsCodY (13). In EfCodY, a salt-bridge can directly link the conformational changes at the Leu binding site to changes in the H1–H2 loop, explaining why Leu alone can activate EfCodY. Interestingly, the salt bridge in EfCodY is made possible by the 4-amino acid insertion in the H1–H2 loop. This insertion closes the distance between the loop and the H3–H4 linker by ∼8 Å. Notably, CodY from Enterococci, Streptococci and Lactococci that do not bind GTP all contain Lys74, Glu26 (or Asp) and a 3–4 amino acid insertion in the H1–H2 loop (16). This raises the possibility that the direct interaction is conserved in CodY from these genera and that the allosteric control of CodY-activity by GTP was gained or lost during bacterial evolution. The reported crystal structures of ligand-free GTP-responsive BsCodY and BcCodY (15,18) are intermediates between inactive and active states, i.e. with the H3-shift already occurred. If the H3-shift is induced by the CodY tetramerization during crystallization or if it indicates a somewhat different mechanism in Bacilli has yet to be addressed. We nevertheless expect CodY to be able to adopt intermediate and activated states even in the absence of ligands, with an equilibrium shift towards the activated state upon ligand-binding.

General rules governing CodY–DNA recognition remain poorly understood because CodY-binding sites display a remarkably high degree of sequence variation. In fact, due to the sequence variation, it was proposed early on that CodY recognizes a specific feature of DNA structure rather than a specific DNA sequence (42). Here, we present the structural basis for the interaction of CodY with overlapping binding sites. These revealed that CodY–DNA recognition is mainly driven by local shape readout of a narrow and deformed A–T rich minor groove. Only two hydrogen bonds between each wHTH motif and half-site DNA contribute to base specificity. The conserved nature of interface-residues, as well as essentially identical DNA-interactions of SaCodY and EfCodY, suggest that the DNA recognition mechanism is common to all CodY proteins.

Genome-wide analysis of CodY-binding sites indicate that CodY-dependent genes largely rely on overlapping binding sites for regulation (20–23). Because of the larger protein-DNA interface in overlapping sites, CodY binds overlapping sites with increased affinity and specificity, which can compensate for weaker and less specific interactions at the individual sites. Individual sites in many overlapping sites of CodY target genes—often containing four or more mismatches (24)—may not bind CodY by themselves, but they do in context of the overlapping site. For example, the overlapping sites in the *hutP* operator of *Bs* contain two and four mismatches, and both sites are required for CodY-mediated regulation *in vivo* (24). With bio-layer interferometry we could show that CodY efficiently only binds to the overlapping site but not to the single sites (Supplementary Figure S12).

However, the affinity of CodY for overlapping sites not only depends on its intrinsic affinities for each individual site but also on cooperativity (21,24). Cooperativity may play an important role in gene regulation by enhancing the responsiveness of target genes towards small changes in activated CodY levels. The presented structures revealed that two CodY dimers assemble onto the overlapping binding sites such that cross-dimer interactions are formed, resulting in DNA-dependent CodY tetramerization. We suggest that binding of the first CodY dimer provides the protein contact surface required to promote binding of the second dimer. Nonetheless, we think that DNA-structure hereby plays an active role. This notion is supported by different minor groove widths of the central and peripheral half sites. In the central, shared half site, the minor grooves are widened at the cross dimer-interactions, thereby linking DNA deformation to cross dimer interactions.

The DNA-bound CodY tetramer allows assembly of even higher-order CodY oligomers through cross-dimer interactions with additional dimers on either side of the tetramer. The assembly of such higher-order CodY oligomers *in vivo* is supported by extended CodY protected regions in regulatory regions predicted to contain more than two overlapping binding sites (24,39). For example, DNase I footprinting in *Bs* identified a CodY protected region in the *ureA* operator that contains three overlapping binding sites with 5, 2 and 3 mismatches respectively to the consensus sequence (24). Here, we showed with electromobility shift assays that this sequence can indeed assemble a DNA-complex containing three SaCodY dimers (Supplementary Figure S13A). A structural model of this complex is shown in Supplementary Figure S13B.

In summary, binding to overlapping sites with less stringent sequence requirements, together with a recognition mechanism largely dictated by shape readout, explains why CodY can recognize target sequences that substantially deviate from the consensus sequence. Binding of CodY to longer sites with little base-specificity provides a potent strategy to facilitate binding site overlaps. These would be necessary in regulatory regions that serve multiple functions, e.g. overlapping operators and promoters or overlapping binding sites for different transcription factors. Overlapping binding sites for different transcription factors allow operators to effectively integrate signals from discrete signal transduction pathways. There are several examples known to date where CodY-binding sites overlap with binding sites of other transcription factors; e.g. PutR and AbrB in *Bs* (43,44). The mechanistic insights into the activity of CodY presented here will guide efforts to uncover the full regulatory potential, a critical stepping-stone in understanding the lifestyle switch to pathogenicity in several important human pathogens.

Other global transcription factors use strategies similar to those of CodY. For example, members of the LysR and IclR families use tetramers to recognize two less-conserved binding sites (45,46). However—unlike CodY—these proteins are tetramers in their free form. For the global repressor Fur an ‘overlapping-dimer binding model’ has been proposed in which Fur cooperatively binds and oligomerizes on operator DNA containing overlapping binding sites (47,48). Thus, our data presented here reveal mechanistic insights that may be relevant to many other bacterial transcription factors and contribute to our general understanding of how these control bacterial behaviour.

## Supplementary Material

gkad512_Supplemental_File

## Data Availability

The atomic coordinates and the structure factors have been deposited with the Protein Data Bank (49) (PDB codes 8c7o for ligand-free SaCodY, 8c7s for SaCodY-Ile-GTP-DNA, 8c7t for ligand-free EfCodY, and 8c7u for EfCodY-Leu-DNA).
