# Supplementary material for: Structural insights into CodY activation and DNA recognition
Source: Nucleic Acids Res. 2023 Jun 16;51(14):7631–48. doi: 10.1093/nar/gkad512 (PMC10415144; doi:10.1093/nar/gkad512)
Supplement: gkad512_Supplemental_File [file gkad512_supplemental_file.pdf]

Supplementary Information

Structural insights into CodY activation and DNA recognition

Tobias Hainzl[1,2*], Mari Bonde[1,3], Fredrik Almqvist[1,2], Jörgen Johansson[2,4,5], A. Elisabeth Sauer-Eriksson[1,2*]

[1]Department of Chemistry, Umeå University, 901 87 Umeå, Sweden

[2]Umeå Centre of Microbial Research (UCMR), Umeå University, Umeå, Sweden

[3]Present address: QureTech Bio, Umeå, Sweden

[4]Department of Molecular Biology, Umeå University, 901 87 Umeå, Sweden

[5]Molecular Infection Medicine, Sweden (MIMS), Umeå University, 901 87 Umeå, Sweden

*corresponding authors

Supplementary Methods

Hydrogen deuterium exchange mass spectrometry (HDX-MS)

The HDX-MS analysis was performed using automated sample preparation on a LEAP H/D-X PAL™ platform interfaced to an LC-MS system, comprising an Ultimate 3000 micro-LC coupled to an Orbitrap Q Exactive Plus MS. Samples constituted 3 µl SaCodY at 70 µM mixed with either 27 µl 25 mM Tris, pH(read) 7.9, or HDX labelling buffer of the same composition prepared in D_2O, pH(read) 7.5. The HDX labelling were carried out for t = 0, 60, 600 seconds at 20°C. The labelling reaction was quenched by dilution of 25 µl labelled sample with 30 µl of 1% trifluoroacetic acid (TFA), 0.4 M Tris(2-carboxyethyl)phosphine hydrochloride (TCEP), and 4 M urea at pH 2.5 and 1°C. 50 µl of the quenched sample was directly injected and subjected to online pepsin digestion at 4°C (in-house immobilized pepsin column, 2.1 x 30 mm). The online digestion and trapping were performed for 4 min using a flow of 50 µl/min of 0.1% formic acid (FA), pH 2.5. The peptides generated by pepsin digestion were subjected to on-line solid phase extraction (SPE) on a PepMap300 C18 trap column (1 mm x 15 mm), and washed with 0.1% FA (buffer A) for 60 s. Thereafter, the trap column was switched in-line with a reversed-phase analytical column, Hypersil GOLD, particle size 1.9 µm, 1 x 50 mm, and separation was performed at 1°C using a gradient of 5-50 % of 95 % acetonitrile/0.1 % FA (buffer B) over 8 minutes and then from 50 to 90% of buffer B for 5 min. Following the separation, the trap and column were equilibrated at 5% organic content, until the next injection. The needle port and sample loop were cleaned three times after each injection with mobile phase 5% methanol/0.1% FA, followed by 90% methanol/0.1% FA and a final wash of 5% methanol/0.1% FA. After each sample and blank injection, the pepsin column was washed by injecting 90 µl of pepsin wash solution 1% FA /4 M urea /5% methanol. In order to minimize carry-over, a full blank was run between each sample injection. Separated peptides were analyzed on a Q Exactive™ Plus MS, equipped with a heated electrospray (HESI) source operated at a capillary temperature of 250°C with sheath gas 12, Aux gas 2 and sweep gas 1 (au). For undeuterated samples (t = 0 s), 1 injection was acquired using data dependent MS/MS HCD for identification of generated peptides. For HDX analysis (all labelled samples and one t = 0 s) MS full scan spectra at setting: resolution 70.000; AGC target 3e6; Max IT 200 ms; and scan range 300-2000 m/z were collected.

Data analysis: The PEAKS Studio X Bioinformatics Solutions Inc. (BSI, Waterloo, Canada) software was used for peptide identification after pepsin digestion of undeuterated samples (i.e., timepoint 0 s). The search was done on a FASTA file with only the SaCodY sequence. Search criteria was a mass error tolerance of 15 ppm and a fragment mass error tolerance of 0.05 Da, allowing for fully unspecific cleavage by pepsin.

Peptides identified by PEAKS with a peptide score value of log P > 25 and no modifications were used to generate a peptide list containing peptide sequence, charge state and retention time for the HDX analysis. HDX data analysis and visualization was performed using HDExaminer, version 3.1.1 (Sierra Analytics Inc., Modesto, US). The analysis allowed only for EX2 and the two first residues of a peptide was assumed unable to hold deuteration. As a full deuteration experiment was not made, full deuteration was set to 75% of max uptake. The presented deuteration data is the average of all high and medium confidence results. The allowed retention time window was ± 0.5 min. Heatmaps settings were uncolored proline with no smoothing allowed. The spectra for all time points were manually inspected; low scoring peptides, obvious outliers, and many peptides where retention time correction could not be made consistent, were removed. The HDX data are reported in Supplementary Table S2, according to recommendation (ref https://www.nature.com/articles/s41592-019-0459-y).

Isothermal titration calorimetry

Wild-type and mutant EfCodY, and the Leu amino acid were prepared in buffer containing 20 mM Tris-HCl pH 8, 400 mM NaCl. In the calorimetry experiments, 500 µM EfCodY was titrated against 80 and 120 mM Leu. Experiments were performed at 25°C using a MicroCal-Auto-ITC200 calorimeter (MicroCal-Malvern) and the standard methods "Plates Prerinse Syringe Clean" and "EDTA". After subtraction of the isotherms obtained by injection of Leu into the buffer alone, the binding isotherms were fitted to the "one set of sites" model implemented in Origin 7 (OriginLab). Experiments were performed in independent triplicates.

Electrophoretic Mobility Shift Assay

In standard mobility shift assays, SaCodY was mixed with DNA at a molar ratio of 8:1 (CodY-protomer/dsDNA) in the presence of 10 mM Ile and 2 mM GTP in a buffer containing 20 mM Tris-HCl pH 8.0, 150 mM NaCl, 0.5 mg/ml t-RNA and incubated at RT for > 1h. Electrophoresis was performed at RT in a 5% polyacrylamide, 10% glycerol, 1 x Tris-acetate-EDTA gel with a 1 x Tris-acetate-EDTA running buffer at 50 V. After electrophoresis, the gel was stained with ethidium bromide.

Supplementary Tables

Supplementary Table S1. Data collection and refinement statistics

	**SaCodY-Apo**	**SaCodY-Ile-GTP-DNA**
**Synchrotron**	MAXIV	ESRF
Beam line	BioMAX[1]	ID23-2[2]
**Data collection and refinement**		
Space group	$P2_1$	$P6_122$
Unit cell parameters (Å,°)	35.80, 163.85, 46.87	104.12, 104.12, 257.26
	89.0, 93.80, 90.0	90.0, 90.0, 120.0
Resolution limits* (Å)	45.0-2.05 (2.12-2.05)	44.70-3.05 (3.16-3.05)
No. of unique reflections	33428 (3376)	16453 (1599)
R_{merge}	0.111 (1.187)	0.379 (2.567)
R_{PIM}	0.074 (0.799)	0.109 (0.733)
$<I / \sigma I>$	5.6 (1.0)	12.1 (1.9)
Half-set correlation CC(1/2)	0.995 (0.44)	0.997 (0.824)
Completeness (%)	99.4 (100.0)	99.9 (100.0)
Multiplicity	3.8 (3.9)	23.2 (24.4)
Wilson B-factor (Å^2)	37.5	69.2
R factor	0.213 (0.343)	0.214 (0.330)
R free	0.256 (0.406)	0.259 (0.354)
No. of protein atoms	4056	4018
No. of DNA atoms	-	1230
No. of GTP/Ile atoms	-	64/18
No. of SO_4^{2-} atoms	100	15
No. of water molecules	100	9
Clash score	2.9	4.0
B-factors (Å^2):		
Protein	52.4	81.2
DNA	-	56.6
GTP/Ile	-	76.2/74.7
SO_4^{2-}	70.6	79.8
Water	48.1	52.0
R.m.s. deviations:		
Bond lengths (Å)	0.005	0.003
Bond angles (°)	0.57	0.61
Ramachandran: Residues in most favored regions (%)	98.6	97.0
Ramachandran: Residues in disallowed regions (%)	0.0	0.0
PDB code	8C7O	8C7S

Estimate of resolution limit is based on CC1/2.

Supplementary Table S1. Data collection and refinement statistics, continue

	EfCodY-Apo	EfCodY-Leu-DNA
**Synchrotron**	ESRF	ESRF
Beam line	ID30B[3]	ID30B[3]
**Data collection and refinement**		
Space group	$P2_12_12_1$	$P2_12_12_1$
Unit cell parameters (Å,°)	32.99, 171.24, 215.24<br>90.0, 90.0, 90.0	86.75, 98.69, 168.36,<br>90.0, 90.0, 90.0
Resolution limits* (Å)	35.11-2.21 (2.29-2.21)	48.79-3.15 (3.26-3.15)
No. of unique reflections	62752 (6079)	25663 (2516)
R_{merge}	0.165 (1.93)	0.249 (3.403)
R_{PIM}	0.077 (0.876)	0.073 (1.010)
$<I / \sigma I>$	7.3 (1.1)	8.5 (1.0)
Half-set correlation CC(1/2)	0.995 (0.501)	0.997 (0.441)
Completeness (%)	99.9 (100.0)	100.0 (100.0)
Multiplicity	6.4 (6.8)	13.2 (13.1)
Wilson B-factor (Å^2)	43	112
R factor	0.237 (0.335)	0.251 (0.399)
R free	0.274 (0.385)	0.279 (0.402)
No. of protein atoms	7518	8097
No. of DNA atoms	-	1211
No. of Leu atoms	-	18
No. of water molecules	141	0
Clash score	5.9	9.7
B-factors (Å^2):		
Protein	77.9	127.9
DNA	-	106.2
Leu	-	119.8
Water	50.4	0
R.m.s. deviations:		
Bond lengths (Å)	0.012	0.005
Bond angles (°)	1.19	0.89
Ramachandran: Residues in most favored regions (%)	98.4	96.8
Ramachandran: Residues in disallowed regions (%)	0.0	0.0
PDB code	8C7T	8C7U

References to beam lines:

[1] Ursby, T., Ahnberg, K., Appio, R., Aurelius, O., Barczyk, A., Bartalesi, A., Bjelcic, M., et al. (2020) BioMAX - the first macromolecular crystallography beamline at MAX IV Laboratory. J Synchrotron Radiat, 27, 1415-1429.

[2] Flot, D., Mairs, T., Giraud, T., Guijarro, M., Lesourd, M., Rey, V., van Brussel, D., Morawe, C., Borel, C. Hignette, O. et al. (2010) The ID23-2 structural biology microfocus beamline at the ESRF. J. Synchrotron Radiat, 17, 107-118.

[3] McCarthy, A.A., Barrett, R., Beteva, A., Caserotto, H., Dobias, F. et al. (2018) ID30B - a versatile beamline for macromolecular crystallography experiments at the ESRF. J Synchrotron Radiat, 25, 1249-1260.

Supplementary Table S2. HDX Experimental details and data

Data Set	ligand-free
HDX reaction details	Undeuterated = 25 mM TBS, pH(read)= 7.1 // HDX labelling at 20°C by 7-fold dilution (85% D_2O)with 25 mM dPBS, pH(read)=7.5 // Quench at 1°C by 1:1 dilution with 4 M Urea, 0.4 M TCEP in 1 % TFA
HDX time course (labelling at 20°C)	60, 600, 3600 ,
HDX control samples*	4 undeuterated controls
# of Peptides	186
Sequence coverage (AA 319-554)	89.8%
Average peptide length / Redundancy	15.8/11.4
Replicate runs (technical) per time point	4
Repeatability *	na
Significant differences; Calculated by HDExaminer, volcano plot significance lines, 95% CI, (Da)	na

* As a full deuteration experiment was not made, full deuteration was set to 75% of max uptake.

Supplementary Figures

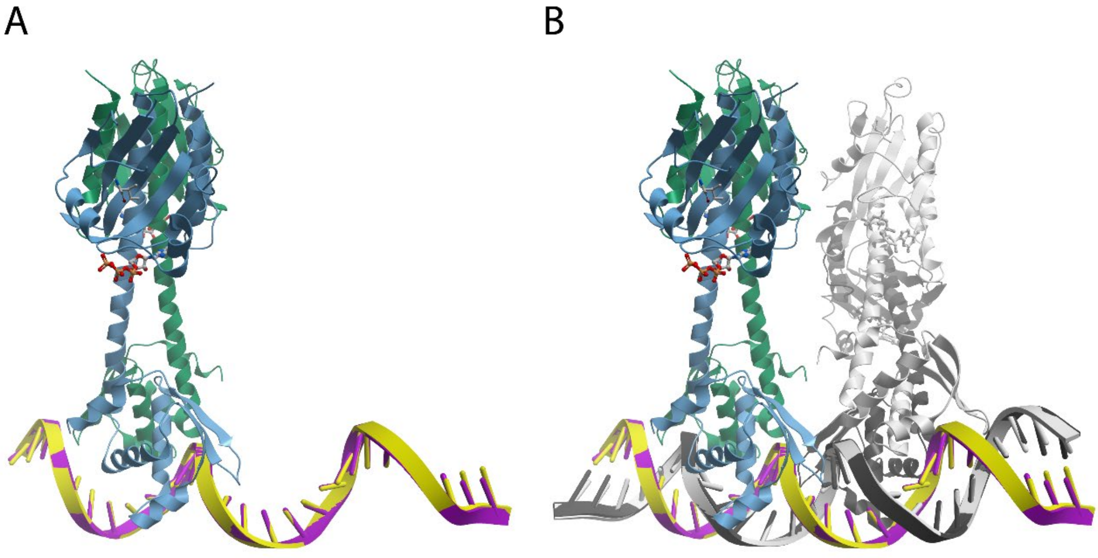

Figure S1. Crystal structure of activated SaCodY-Ile-GTP in complex with DNA. (**A**) The asymmetric unit of the complex contains one CodY dimer and one ssDNA comprising both the DNA template and the complementary strands, each refined at half occupancy. (**B**) The biological assembly of the structure. The symmetry-related structure is shown in light and dark gray.

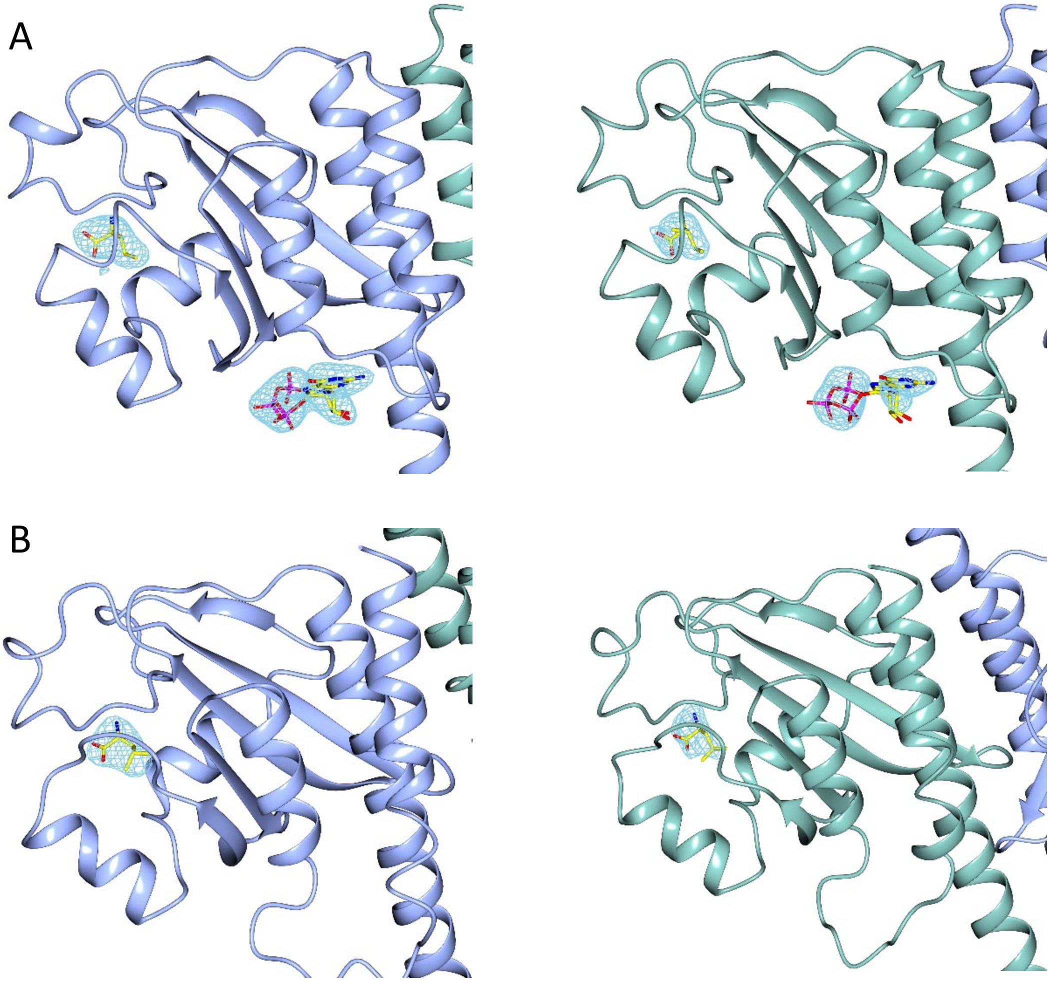

Figure S2. Representative polder electron density maps covering ligands bound to the refined model of SaCodY-DNA and EfCodY-DNA. Maps are contoured at five times the RMS value of the map. (**A**) SaCodY-DNA: Ile and GTP binding to protomers A and B. Calculated correlation coefficients (CC(1,3)) for the ligands: A-Ile, 0.93; A-GTP, 0.91; B-Ile, 0.88; and B-GTP, 0.88. (**B**) EfCodY-DNA: Leu binding to protomers A and B. Calculated CC(1,3) for the ligands: A-Leu, 0.92; and B-Leu, 0.85. Protomers A and B are colored ice-blue and sea-green, respectively.

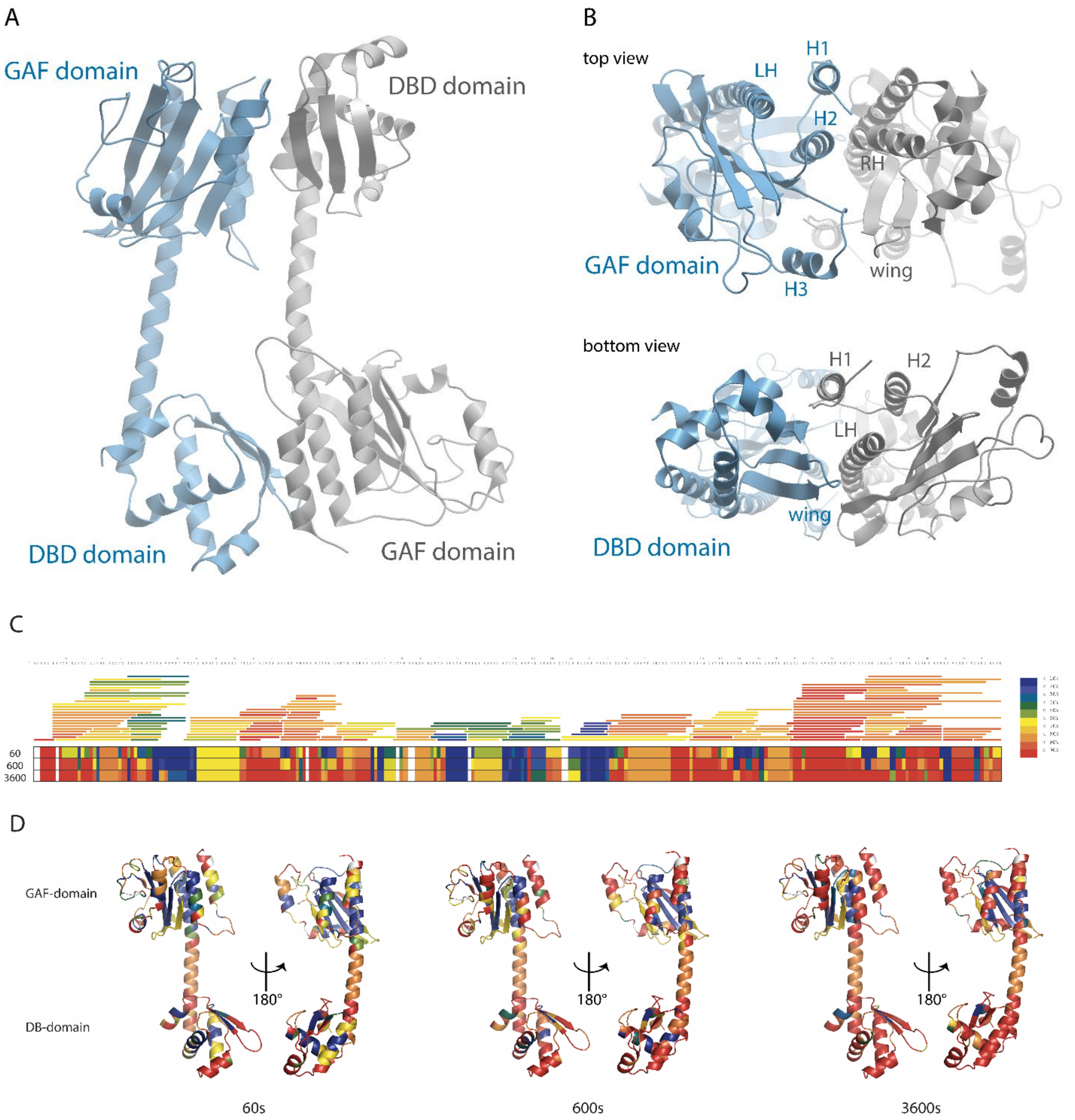

Figure S3. The DBD domain in ligand-free SaCodY is flexible. Ribbon representations of the side view (**A**) and top and bottom views (**B**) of the head-to-tail arrangement of SaCodY in the crystal. (**C**) HDX-MS heatmap for SaCodY (2 mg/ml) showing deuterium uptake at the different labelling times (1 min, 10 min, and 60 min), color-coded based on a least-squares calculation of observed deuterium uptake without smoothing (see color key at right). A cold color (slower exchange) indicates protection or secondary structure, while a warm color represents fast exchange, indicating unstructured regions. The bars above the heatmap show the sequence coverage (186 peptides for 90% coverage) and are color-coded for uptake averaged over all time points. The sequence for SaCodY is indicated on the top. (D) Overlay of the heatmap data on the ligand-free SaCodY structure shows that the DBD domain is largely unstructured.

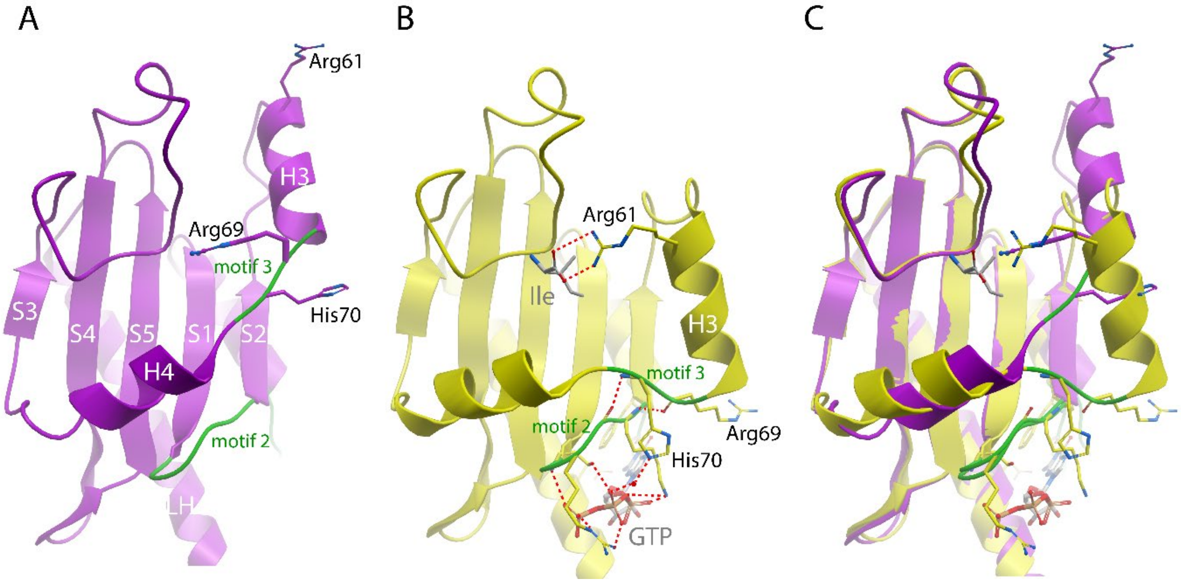

Figure S4. Conformational changes in the GAF domain of SaCodY upon Ile/GTP binding. Ribbon representations with selected residues shown as sticks. (**A**) The ligand-free GAF domain, (**B**) the Ile/GTP-bound GAF domain, and (**C**) superimposition of the two based on residues 25-155. GTP-binding motifs 2 and 3 are highlighted in green. GTP-binding motif 1 is not visible in this orientation.

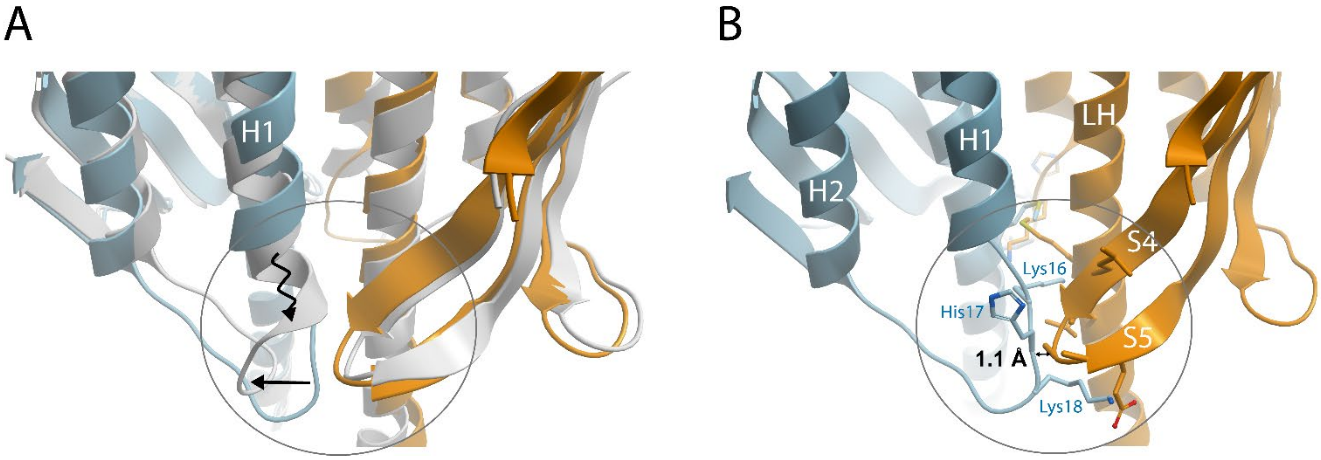

Figure S5. Steric clashes prevent dimer formation of the ligand-free monomeric structure of SaCodY. (**A**) Two copies (blue and orange) of the monomeric structure of the GAF domain present in ligand-free SaCodY are overlayed on protomer A and B (colored grey) of the dimeric GAF domain structure present in the Ile/GTP-bound SaCodY. The overlay is based on residues 85-135. Ligand-binding induces an extension of H1 (indicated by the wavy arrow) that moves residues Gln15-Lys18 (straight arrow) to allow dimer formation. (**B**) Shows how the conformation of the H1-H2 loop in the monomeric structure would sterically clash with the linker helix (LH) and the S3-S4 loop of protomer B in a dimeric structure. The sites where clashes occur are encircled.

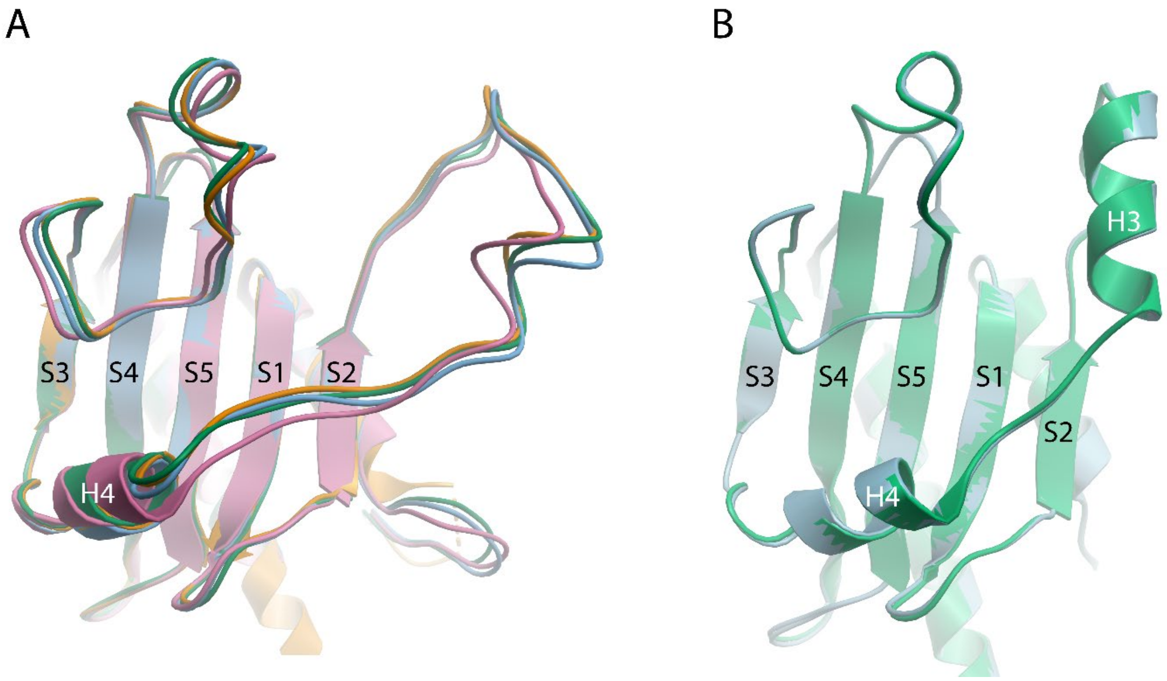

Figure S6. Helix H3 is absent in the GAF domain of ligand-free EfCodY. (A) Ribbon representation of the superimposed GAF domains of all four protomers in the asymmetric unit of the ligand-free EfCodY crystals. In all protomers, residues 58-79 between S2 and H4 form an identical extended loop structure although their crystal contacts are not identical, arguing that helix H3 does not exist in ligand-free EfCodY. The ribbon representation of protomer A is colored blue, protomer B green, protomer C orange, and protomer D purple. The overlay was generated by superimposition of residues 90-140. (**B**) Ribbon drawing of the two monomers in the asymmetric unit of the ligand-free SaCodY crystals. The overlay was generated by superimposition of residues 85-135.

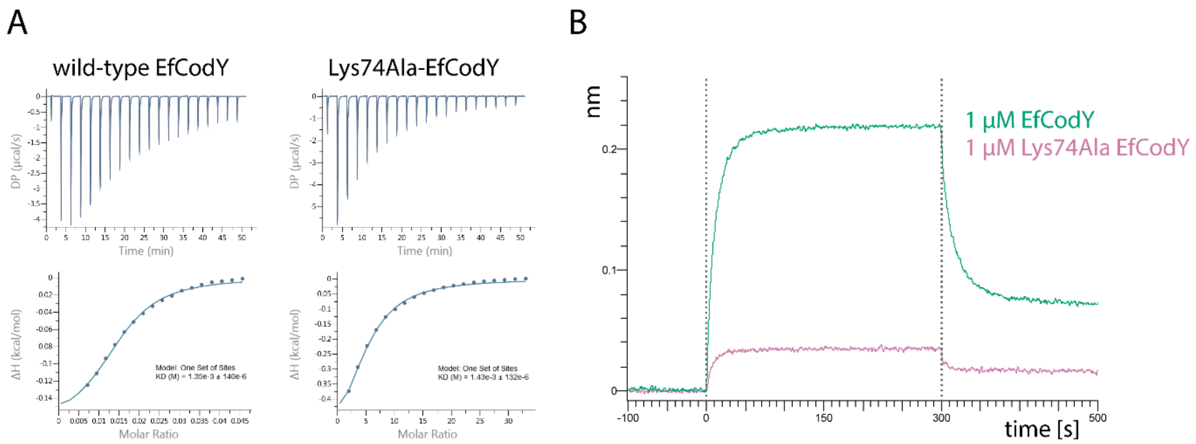

Figure S7. Lys74 in EfCodY is critical for propagating the Leu-induced structural changes. **(A)** Isothermal titration calorimetry thermograms and corresponding fitted curves generated for the injection of Leu into samples of wild-type and Lys74Ala-mutant EfCodY showing that Leu-binding is not impeded in the mutant. For the wild-type and the mutant, the curves determined KD values of 1.3-1.4 mM, consistent with the moderate affinities previously reported for *Bs* (1). (**B**) Representative bio-layer interferometry sensorgrams of the interaction of wild-type EfCodY and Lys74Ala-mutant EfCodY with the *hutP* operator sequence in the presence of 10 mM Leu, showing that DNA-binding is strongly reduced for the mutant compared with the wild-type.

1. Shivers, R.P. and Sonenshein, A.L. (2004) Activation of the Bacillus subtilis global regulator CodY by direct interaction with branched-chain amino acids. *Mol Microbiol*, **53**, 599-611.

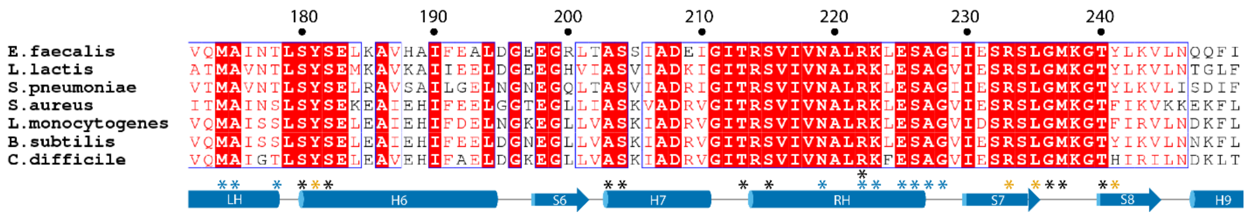

Figure S8. Sequence alignment of CodY DBD domains of selected species from different bacterial genera. The numbering corresponds to SaCodY. The secondary structure is indicated below the alignment. Black stars denote residues forming hydrogen bonds with the DNA, blue stars denote residues stabilizing the dimer interface, and orange stars denote residues stabilizing the cross-dimer interface.

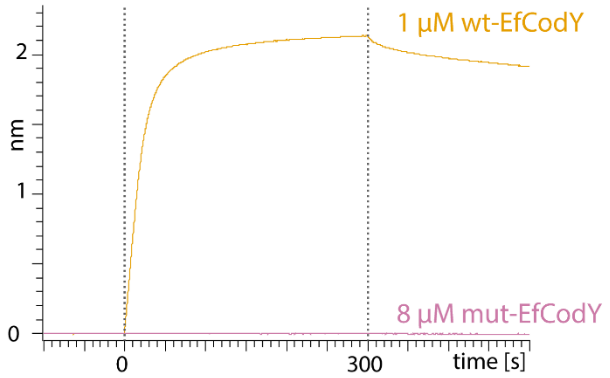

Figure S9. Residues mediating the cross-dimer interface are required for DNA-binding. Representative bio-layer interferometry sensorgrams of the interaction of 1 µM wild-type EfCodY and 8 µM quadruple mutant EfCodY (Tyr186Ala, Arg238Ala, Leu240Ala, Tyr246Ala) with the 24-nt sequence-optimized overlapping binding sites in the presence of 10 mM Leu. Even at 8 µM, the EfCodY quadruple mutant shows no DNA-binding.

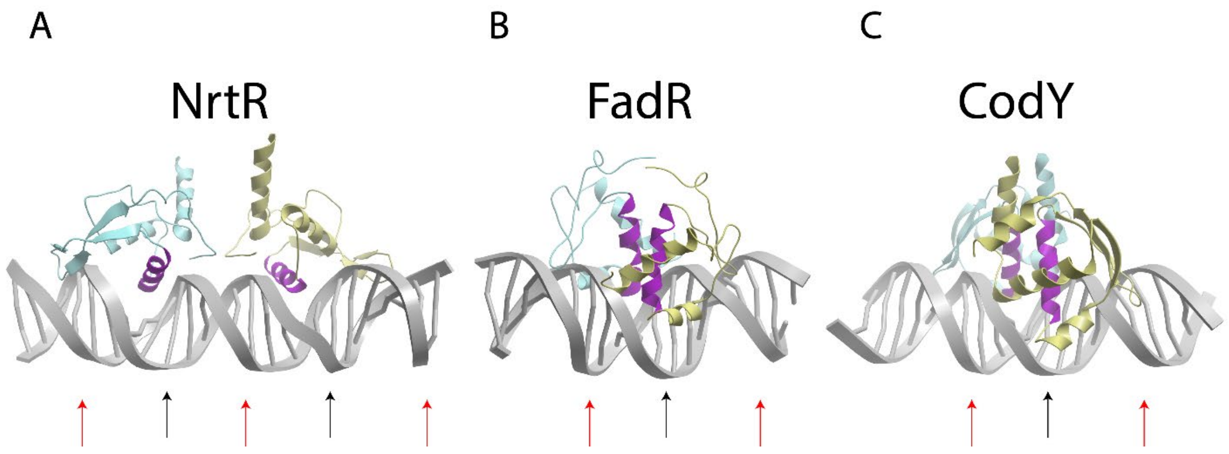

Figure S10. Ribbon representation of canonical and non-canonical wHTH-DNA interactions. (**A**) Canonical wHTH-DNA interaction exemplified by the NrtR dimer (PDB code 3gz6). (**B**) Non-canonical wHTH-DNA interaction of the FadR dimer and the (**C**) CodY dimer. Recognition helices are shown in purple, and the major and minor DNA grooves are indicated by black and red arrows, respectively.

A

```
                                AATTTTCWGAAAATT
                                         AATTTTCWGAAAATT
Consensus        CCGCCGGATAATTTTCAGAATTTTCAGAAAATTTAGCCG
Consensus T>A    CCGCCGGATAATTTACAGTATTTACAGTAAATTTAGCCG
Consensus T>G    CCGCCGGATAATTTGCAGCATTTGCAGCAAATTTAGCCG
```

B

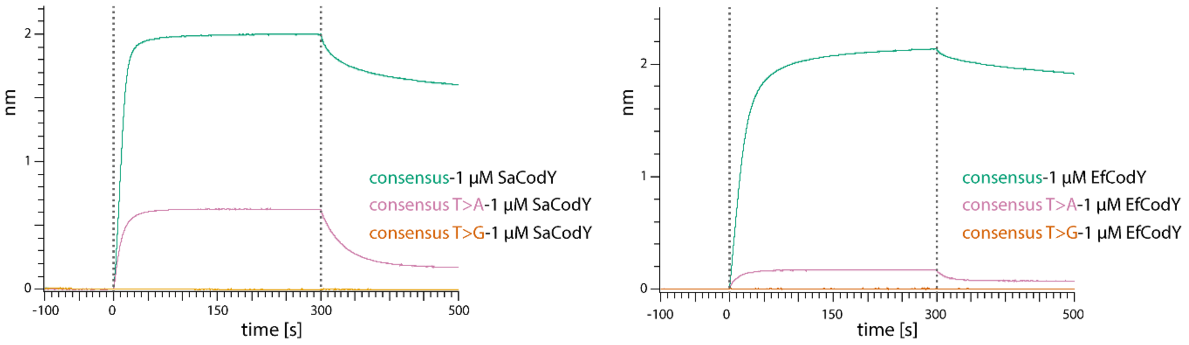

Figure S11. **Thymine bases at positions 6, 15', 15, and 6' are required for efficient CodY-DNA recognition.** (**A**) DNA sequences used in the bio-layer interferometry assays: the sequence-optimized overlapping binding sites (consensus) and the overlapping binding sites with mutations of T-A to A-T or G-C at positions 6, 10, 15, and 19 (consensus T>A and consensus T>G; mutations are red underlaid). The binding sites are highlighted in red; the consensus sequence for the single site is shown above the sequence-optimized binding sites. (**B**) Representative sensorgrams of the interaction of 1 µM SaCodY (in the presence of 10 mM Ile and 2 mM GTP) and EfCodY (in the presence of 10 mM Leu) with the DNA sequences. Weak binding is detected for the overlapping sites containing the T-A to A-T mutations, while no binding is detected for the overlapping sites containing the T-A to G-C mutations.

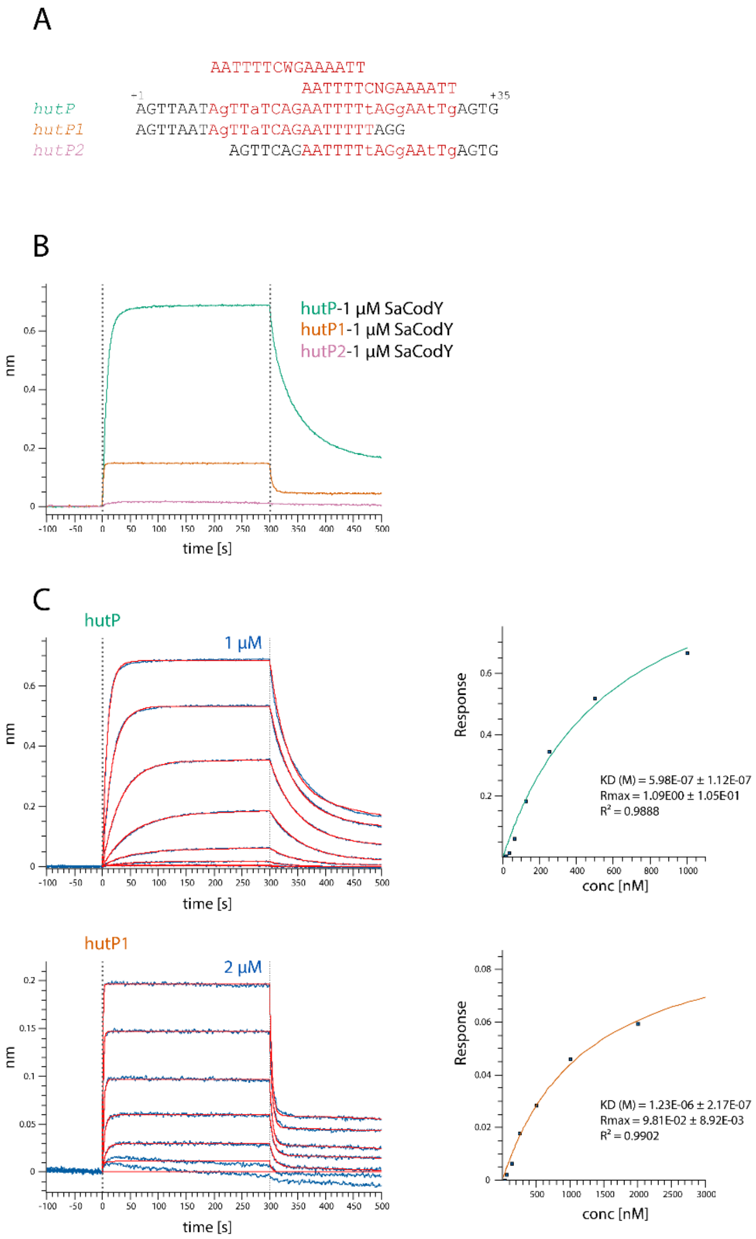

Figure S12. SaCodY binds single sites with low affinity. (**A**) Sequences of the *hutP* operator in *Bs* used in the bio-layer interferometry assay: overlapping binding sites (*hutP* nt position +8 to +31) and its single binding sites (*hutP1* nt +8 to +22 and *hutP2* nt +17 to +31). Binding sites are highlighted in red; mismatches to the consensus are shown in lowercase (*hutP1* has two mismatches, *hutP2* has four mismatches); the consensus sequence is shown above the *hutP* operator sequences. (**B**) Representative bio-layer interferometry sensorgrams of the interaction of 1 µM SaCodY with immobilized *hutP* operator fragments *hutP*, *hutP1*, and *hutP2*. (**C**) Sensorgrams of a twofold dilution series of SaCodY interacting with operator fragments *hutP* and *hutP1*. The highest concentration of the series is indicated. The curve fittings are depicted in red. The affinity plot response vs. SaCodY concentration—derived from the curve fittings—determined a KD of 598 nM for *hutP* and a KD of 1.23 µM for *hutP1*. For *hutP2* no binding was detected at 1 µM SaCodY.

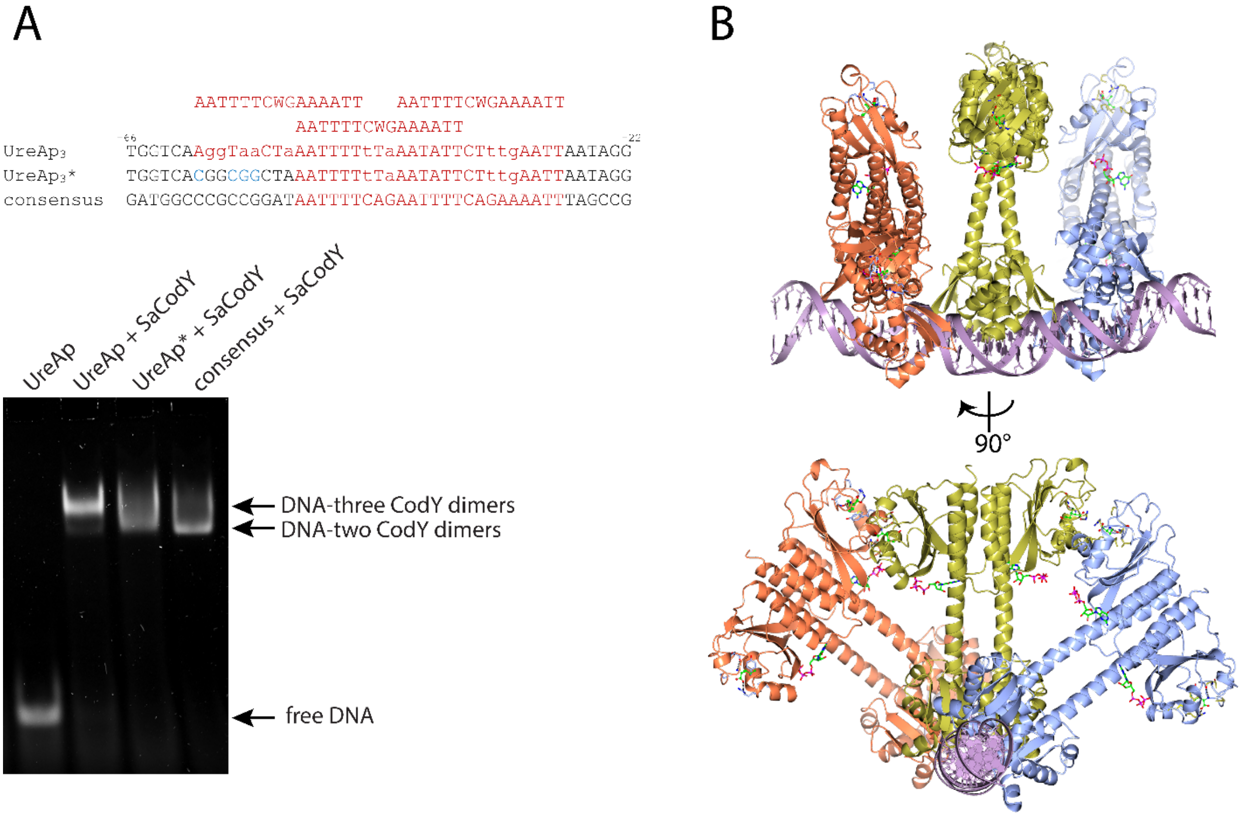

Figure S13. CodY can form higher-order oligomers on operator DNA. (**A**) **Top**: The native UreAp operator sequence in *Bs* (nt position -66 to -22) is predicted to contain three overlapping binding sites (red) with respectively 5, 2, and 3 mismatches (lower letter) to the consensus sequence. In order to disrupt CodY-binding to one of the putative binding sites, we substituted A-T base pairs in the AT sequence of the 5' half-site with G-C base pairs (UreAp*, mutated bases colored blue). Substitution of A-T base pairs in AT minor grooves has been shown to disrupt narrow width profiles (1)—a key determinant of DNA shape recognition by CodY. The consensus DNA contains overlapping binding sites with optimized sequence similarity to the consensus sequence (identical to the DNA in the crystal structures but extended to 45 nt with G-C base pairs). The consensus sequence for the single site is shown above the UreAp operator sequence **Bottom**: Electrophoretic mobility shift assay of the different 45-nt DNAs with SaCodY shows distinctly shifted DNA bands corresponding to three and two dimers bound to the DNA. (**B**) Structural model of three SaCodY dimers bound to DNA.

1. Hancock, S.P., Ghane, T., Cascio, D., Rohs, R., Di Felice, R. and Johnson, R.C. (2013) Control of DNA minor groove width and Fis protein binding by the purine 2-amino group. Nucleic Acids Res, 41, 6750-6760.
